# Sensory Perturbations from Hindlimb Cutaneous Afferents Generate Coordinated Functional Responses in All Four Limbs during Locomotion in Intact Cats

**DOI:** 10.1523/ENEURO.0178-22.2022

**Published:** 2022-12-16

**Authors:** Angèle N. Merlet, Pierre Jéhannin, Stephen Mari, Charly G. Lecomte, Johannie Audet, Jonathan Harnie, Ilya A. Rybak, Boris I. Prilutsky, Alain Frigon

**Affiliations:** 1 Department of Pharmacology-Physiology, Faculty of Medicine and Health Sciences, Centre de Recherche du Centre Hospitalier Universitaire de Sherbrooke, Université de Sherbrooke, Sherbrooke, Quebec J1H 5N4, Canada; 2 Department of Neurobiology and Anatomy, College of Medicine, Drexel University, Philadelphia, PA 19129; 3 School of Biological Sciences, Georgia Institute of Technology, Atlanta, GA 30332

**Keywords:** locomotion, stumbling corrective reaction, sensory feedback, cutaneous

## Abstract

Coordinating the four limbs is an important feature of terrestrial mammalian locomotion. When the foot dorsum contacts an obstacle, cutaneous mechanoreceptors send afferent signals to the spinal cord to elicit coordinated reflex responses in the four limbs to ensure dynamic balance and forward progression. To determine how the locomotor pattern of all four limbs changes in response to a sensory perturbation evoked by activating cutaneous afferents from one hindlimb, we electrically stimulated the superficial peroneal (SP) nerve with a relatively long train at four different phases (mid-stance, stance-to-swing transition, mid-swing, and swing-to-stance transition) of the hindlimb cycle in seven adult cats. The largest functional effects of the stimulation were found at mid-swing and at the stance-to-swing transition with several changes in the ipsilateral hindlimb, such as increased activity in muscles that flex the knee and hip joints, increased joint flexion and toe height, increased stride/step lengths and increased swing duration. We also observed several changes in support periods to shift support from the stimulated hindlimb to the other three limbs. The same stimulation applied at mid-stance and the swing-to-stance transition produced more subtle changes in the pattern. We observed no changes in stride and step lengths in the ipsilateral hindlimb with stimulation in these phases. We did observe some slightly greater flexions at the knee and ankle joints with stimulation at mid-stance and a reduction in double support periods and increase in triple support. Our results show that correcting or preventing stumbling involves functional contributions from all four limbs.

## Significance Statement

The skin contains mechanoreceptors that, when activated, send afferent signals to the spinal cord, signaling a perturbation. For example, when the foot dorsum hits an obstacle during the swing phase, cutaneous inputs trigger a functional reflex response in all four limbs to rapidly move the leg away from and over the stimulus or obstacle to ensure dynamic balance and forward progression. Here, we investigate the locomotor pattern of all four limbs after cutaneous nerve stimulation of one hindpaw at four different phases of the step cycle during quadrupedal treadmill locomotion. Stimulating cutaneous afferents innervating the foot dorsum generates functional responses involving the whole-body. These responses are phase dependent and serve to correct or prevent stumbling.

## Introduction

Proprioceptive and tactile feedback regulates the locomotor pattern by interacting with central neural circuits (for recent review, see Frigon et al., 2021). For instance, cutaneous mechanoreceptors send afferent signals to the spinal cord and influence motor output during real and fictive locomotion (Rossignol et al., 2006; Grillner and El Manira, 2020; Frigon et al., 2021). During real locomotion, when the foot dorsum hits an obstacle during the swing phase, cutaneous inputs trigger a functional reflex response called the stumbling corrective reaction to rapidly move the leg away from and over the stimulus or obstacle. The stumbling corrective reaction has been observed in many mammals, including intact and spinal-transected cats (Forssberg et al., 1977; Prochazka et al., 1978; Forssberg, 1979; Wand et al., 1980; Buford and Smith, 1993; Quevedo et al., 2005a; McVea and Pearson, 2007) and mice (Mayer and Akay, 2018), as well as in human infants, adults and the elderly (Schillings et al., 1996, 2000, 2005; Van Wezel et al., 1997; Zehr et al., 1997; Haridas and Zehr, 2003; Lam et al., 2003; Potocanac et al., 2016).

Stimulating nerves of the foot, such as the superficial peroneal (SP) nerve that innervates the foot dorsum, elicits specific reflex responses in the ipsilateral (stimulated limb) and contralateral (opposite limb of same girdle) legs/hindlimbs but also in the homolateral (limb of the other girdle on same side) and diagonal (limb of the other girdle on opposite side) arms/forelimbs in cats and humans during locomotion (Haridas and Zehr, 2003; Hurteau et al., 2018; Pearcey and Zehr, 2019). Thus, the stumbling corrective reaction is part of a whole-body response that coordinates activity in all four limbs. Coordinating the four limbs is a fundamental requirement for quadrupedal locomotion in mammals and bipedal locomotion in humans to maintain balance in an ever-changing environment (for review, see Dietz, 2002; Dietz and Michel, 2009; Zehr et al., 2016; Frigon, 2017). To be functionally appropriate, responses evoked by cutaneous inputs during locomotion are strongly modulated by phase (Miller et al., 1977; Buford and Smith, 1993; Van Wezel et al., 1997; Zehr et al., 1997; Hurteau et al., 2018). For example, stimulating the SP nerve during the stance or extension phase does not elicit limb withdrawal. Instead, it evokes responses that prevent stumbling by strengthening extensor activity in the stimulated limb (Forssberg, 1979; Quevedo et al., 2005a). This has been termed the stumbling preventive reaction.

Studies have shown that stimulating nerves of the foot mainly alters the structure of the step cycle of the ipsilateral hindlimb/leg in cats and humans when applied during swing (Duysens and Pearson, 1976; Forssberg et al., 1977; Forssberg, 1979; Buford and Smith, 1993; Schillings et al., 1996; Zehr et al., 1997). For example, the ipsilateral hindlimb swing phase is prolonged along with contralateral stance. The same stimulation during stance of the ipsilateral hindlimb produces weaker effects on the structure of the step cycle. Some studies also observed temporal adjustments in the cycle following stimulation (Forssberg et al., 1977; Buford and Smith, 1993). Although we know that cutaneous inputs signaling a perturbation are distributed to the four limbs during quadrupedal locomotion and phase-modulated, we do not know how they affect the spatiotemporal structure of the interlimb pattern at different times during the step cycle. A recent study showed phase-dependent reflex responses in flexor and extensor muscles of the hindlimb stimulated and in muscles of the other three limbs during treadmill locomotion in cats (Hurteau et al., 2018). In that study, however, the stimulation parameters (three 0.2-ms pulses at an intensity just above the motor threshold) were selected to limit perturbing the stimulated limb. In the present study, we stimulated the SP nerve with a longer train (25 0.2-ms pulses) to induce a noticeable perturbation of the stimulated limb during mid-swing. We used the same stimulation intensity in three other parts of the cycle to assess phase-dependency. Based on the responses observed by Hurteau et al. (2018), we anticipate phase-dependent changes in cycle and phase durations, support periods, as well as stride and step lengths in the forelimbs and hindlimbs with a cutaneous perturbation of the hindlimb. We predict that the effects on the locomotor pattern of the four limbs will be greater with stimulation during the swing phase, as stimulating the SP nerve or foot dorsum during stance has no or weak effects on the structure of the step cycle for the hindlimbs/legs (Forssberg, 1979; Zehr et al., 1997). Therefore, the purpose of this study was to characterize functional changes in the locomotor pattern of all four limbs after a cutaneous nerve stimulation that mimics a mechanical stimulus of one hindpaw at four different phases of the step cycle during quadrupedal treadmill locomotion. Additionally, we used recordings of muscle activity [electromyography (EMG)] in the four limbs to provide insight on how the central nervous system contributes to functional changes.

## Materials and Methods

### Ethical approval

The Animal Care Committee of the Université de Sherbrooke approved all procedures in accordance with policies and directives of the Canadian Council on Animal Care (Protocol 442-18). We obtained the current dataset from seven adult cats (three females and four males) with a mass between 3.5 and 6.9 kg. Before and after experiments, cats were housed and fed in a dedicated room within the animal care facility of the Faculty of Medicine and Health Sciences at the Université de Sherbrooke. As part of our effort to maximize the scientific output of each animal, all animals participated in other studies to answer different scientific questions, some of which have been published (Lecomte et al., 2021). For instance, cats participated in experiments where they walked on a split-belt treadmill at different speeds and left-right speed differences with or without stimulating peripheral nerves, such as SP, superficial radial and distal tibial. They also performed quadrupedal and hindlimb-only locomotion in the forward and backward directions (Harnie et al., 2021, 2022). Cats also stepped on a walkway where they negotiated obstacles and stepped along a circular path. In all seven cats of the present study, we performed a lateral spinal hemisection at mid-thoracic levels and performed the same data collection as in the intact state. We collected the data presented here three to six weeks after implanting electrodes during a single experimental session.

### General surgical procedures

We performed the implantation surgery under aseptic conditions in an operating room with sterilized equipment, as previously described (Hurteau et al., 2017; Harnie et al., 2019, 2021; Merlet et al., 2020, 2021). Briefly, cats first received an intramuscular injection containing butorphanol (0.4 mg/kg), acepromazine (0.1 mg/kg), glycopyrrolate (0.01 mg/kg), and ketamine/diazepam (0.11 ml/kg in a 1:1 ratio, i.m.) for sedation and induction. Cats were then anesthetized with isoflurane (1.5–3%) using a mask and then intubated with a flexible endotracheal tube. Anesthesia was maintained by adjusting isoflurane concentration as needed (1.5–3%). After surgery, we injected an antibiotic (cefovecin, 0.1 ml/kg) subcutaneously and taped a transdermal fentanyl patch (25 μg/h) to the back of the animal 2–3 cm rostral to the base of the tail for prolonged analgesia (removed after 5–7 d). We also injected buprenorphine (0.01 mg/kg), a fast-acting analgesic, subcutaneously at the end of the surgery and ∼7 h later. After each surgery, we placed the cats in an incubator until they regained consciousness. At the conclusion of the experiments, a lethal dose of pentobarbital was administered through the left or right cephalic vein under isoflurane anesthesia.

### Implantation procedure

To record EMG, we directed pairs of Teflon-insulated multistrain fine wires (AS633; Cooner Wire) subcutaneously from two head-mounted 34-pin connectors (Omnetics) that were sewn into the belly of selected forelimb and hindlimb muscles for bipolar recordings, with 1–2 mm of insulation stripped from each wire. The head connector was secured to the skull using dental acrylic and four to six screws. During surgery, we verified electrode placement by electrically stimulating each muscle through the appropriate head connector channel and assessed the muscle contraction. The current data set includes EMG from the following muscles bilaterally: biceps brachii (BB, elbow flexor), biceps femoris anterior (BFA; hip extensor), biceps femoris posterior (BFP; knee flexor/hip extensor), extensor carpi ulnaris (ECU; wrist extensor), lateral gastrocnemius (LG; ankle extensor/knee flexor), anterior sartorius (SRT; hip flexor/knee extensor), soleus (SOL; ankle extensor), semitendinosus (ST; knee flexor/hip extensor), the long head of the triceps brachii (TRI; elbow and shoulder extensor), vastus lateralis (VL; knee extensor). Table 1 provides the muscles analyzed for each cat. For bipolar nerve stimulation, pairs of Teflon-insulated multistrain fine wires (AS633; Cooner Wire) were passed through a silicon tubing. A horizontal slit was made in the tubing and wires within the tubing were stripped of their insulation. The ends protruding through the cuff were knotted to hold the wires in place and glued. The ends of the wires away from the cuff were inserted into an Omnetic connector for bipolar nerve stimulation. Cuff electrodes were directed subcutaneously from head-mounted connectors and placed around the left and right SP nerves at ankle level. At this level, the SP nerve is purely cutaneous (Bernard et al., 2007).

**Table 1:** Muscles analyzed for each cat

	Cat AR	Cat GR	Cat JA	Cat KA	Cat KI	Cat MI	Cat TO
SRT	X	X	X	X	X	X	X
BFP	X	X		X		X	X
ST	X	X	X	X		X	
BFA	X	X		X		X	X
VL	X	X		X		X	X
LG	X	X		X	X	X	X
SOL	X	X	X	X		X	X
BB		X		X	X	X	X
TRI	X	X	X	X	X	X	X
ECU	X		X	X	X		X

The table provides the muscles analyzed for each cat (*n* = 7). SRT, anterior sartorius (*n* = 7 cats); BFP, biceps femoris posterior (*n* = 5 cats); ST, semitendinosus (*n* = 6 cats); BFA, biceps femoris anterior (*n* = 5 cats); VL, vastus lateralis (*n* = 5 cats); LG, lateral gastrocnemius (*n* = 6 cats); SOL, soleus (*n* = 6 cats); BB, biceps brachii (*n* = 6 cats); TRI, the long head of the triceps brachii (*n* = 7 cats); ECU, extensor carpi ulnaris (*n* = 5 cats).

### Experimental protocol

**Figure 1.**
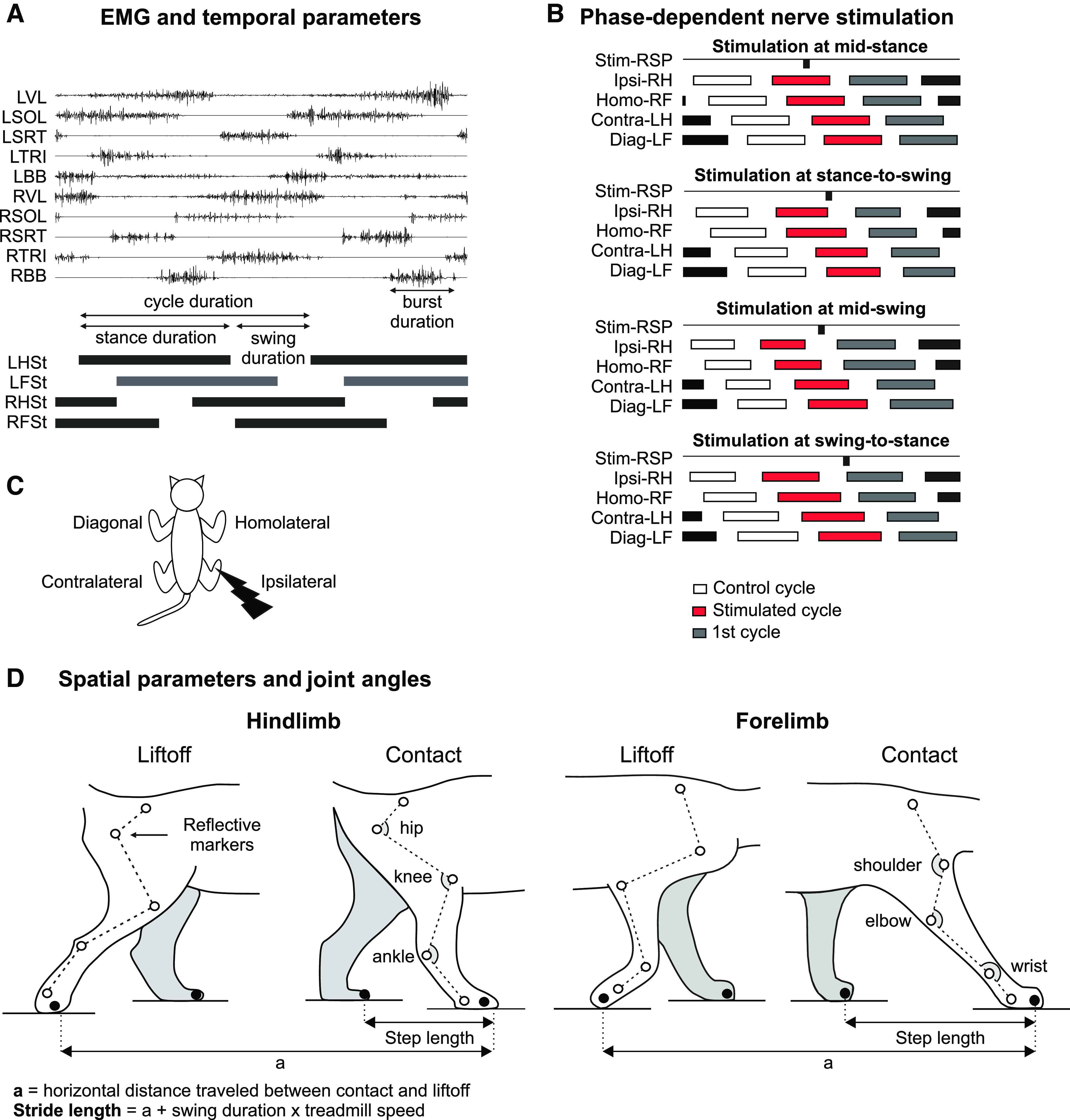
Experimental design. ***A***, The figure shows electromyography (EMG) from selected muscles and stance phases of the four limbs during locomotion in a single cat along with some temporal measures. ***B***, The superficial peroneal (SP) nerve was electrically stimulated at varying delays relative to an ipsilateral hindlimb extensor burst onset to evoke responses at four phases of the step cycle. We measured variables in three consecutive cycles. ***C***, The different limbs are defined based on the stimulation. The ipsilateral and contralateral limbs are the stimulated and opposite hindlimbs, respectively. The homolateral and diagonal limbs are the forelimbs on the same and opposite sides of the stimulated hindlimb, respectively. ***D***, We placed reflective markers on bony landmarks and measured spatial parameters and joint angles. BB, biceps brachii; Contra, contralateral; Diag, diagonal; F, forelimb; H, hindlimb; Homo, homolateral; Ipsi, ipsilateral; L, left; R, right; SOL, soleus; SRT, anterior sartorius; St, stance TRI, long head of triceps brachii; VL, vastus lateralis.

Figure 1 schematically illustrates the experimental set-up and protocol. Cats performed experiments on a treadmill with two independently controlled running surfaces 120 cm long and 30 cm wide (Bertec). Cats were initially trained to step on the treadmill at various tied-belt (both belts at same speed) speeds using food and affection as reward. During experiments, we stimulated the left or right SP nerve while cats stepped at speeds of 0.3 to 0.5 m/s. The speed varied between cats because we aimed to use the slowest speed at which animals could maintain a consistent locomotor pattern. We electrically stimulated the SP nerve with a Grass S88 Stimulator at an intensity of 1.2 times the motor threshold, defined as the voltage that elicited a small consistent short-latency (8–10 ms) excitatory response in an ipsilateral flexor, such as ST. We determined motor threshold during mid-swing and used the same stimulation intensity in the other phases. Each stimulation consisted of a train of 25 impulses (0.2-ms duration, 300-Hz frequency) for a total train duration of 88 ms. These stimulation parameters are similar to those used to describe the neuronal circuits involved in the stumbling corrective reaction with SP nerve stimulation in decerebrate curarized cats during fictive locomotion (Quevedo et al., 2005a,b). The correspondence of these stimulation parameters to natural stimuli is not easy to demonstrate because when the cat’s foot dorsum contacts an obstacle, hair cells are first deflected followed by mechanoreceptors in superficial and deep layers of the skin. The stumbling corrective reaction can result from a combined activation of these different receptors. However, Buford and Smith (1993) reported that the foot dorsum made contact for ∼100 ms with the force-sensitive rod, which is close to our 88-ms train. We delivered ∼60 stimuli pseudo-randomly every five to six step cycles and analyzed three consecutive cycles: the cycle preceding stimulation, the stimulated cycle and the cycle following stimulation. Stimuli were delivered at varying delays relative to an ipsilateral hindlimb extensor burst onset to evoke responses at four phases of the step cycle: mid-stance, stance-to-swing transition, mid-swing and the swing-to-stance transition. We obtained ∼5–20 stimuli per locomotor phase for all cats.

### Data acquisition and analysis

#### Temporal variables

During experiments, two cameras (Basler AcA640-100 gm) captured videos of the left and right sides at 60 frames/s with a spatial resolution of 640 × 480 pixels. A custom-made Labview program acquired images and synchronized them. We used custom-made software to analyze videos offline at 60 frames/s. By visual inspection, we determined, for all four limbs, stance onset as the first frame where the paw made visible contact with the treadmill surface and stance offset as the most caudal displacement of the toes. For all four limbs, we measured cycle duration from successive stance onsets, whereas stance duration corresponded to the interval of time from stance onset to offset (Fig. 1*A*). We measured swing duration as cycle duration minus stance duration (Fig. 1*A*). From the events of the ipsilateral limb (i.e., contact and liftoff of the paw), we determined by visual inspection if the stimulus fell in one of the four phases (Fig. 1*B*). When the stimulus occurred outside of the defined ranges, we excluded it from the analysis (Fig. 1*B*). Figure 1*C* shows the four limbs in relation to the stimulated hindlimb.

#### Spatial variables

Figure 1*D* shows spatial parameters that we measured from kinematic events. Reflective markers were placed on the skin over bony landmarks on the scapula, minor tubercle of the humerus, elbow, wrist, metacarpophalangeal joint and at the tips of the forepaws for the forelimbs and over the iliac crest, greater trochanter, knee, lateral malleolus, metatarsophalangeal joint and at the tips of the hindpaws for the hindlimbs (Fig. 1*D*). To calculate spatial variables, we obtained *x* and *y* coordinates of each marker using DeepLabCut, an open-source software package that uses deep learning for motion tracking (Mathis et al., 2018; Nath et al., 2019), as we recently described in the cat (Lecomte et al., 2021). We measured step lengths, defined as the distance between the leading and trailing limbs at stance onset of the leading limb for the forelimbs and hindlimbs (Hoogkamer et al., 2014; Fig. 1*D*). Stride lengths were measured as the horizontal distance between stance onset and offset of a given limb added to the distance traveled by the treadmill during the swing phase, which was calculated by multiplying swing duration by treadmill speed (Courtine et al., 2005; Thibaudier and Frigon, 2014; Dambreville et al., 2015; Harnie et al., 2018; Fig. 1*D*). We measured total angular excursion (max–min values) for the hip, knee and ankle joints across the cycle for the hindlimbs (Fig. 1*D*). We also calculated the maximum height above the supporting surface of the hip, knee and toes for the hindlimbs, and shoulder, elbow and toes for the forelimbs across the cycle.

#### Electromyography

EMG signals were preamplified (10×, custom-made system), bandpass filtered (30–1000 Hz) and amplified (100–5000×) using a 16-channel amplifier (AM Systems Model 3500). EMG data were digitized (5000 Hz) with a National Instruments card (NI 6032E), acquired with custom-made acquisition software, and stored on a computer. To quantify EMG bursts, we selected three flexor (BFP, *n* = 5; SRT, *n* = 7; and ST, *n* = 6) and four extensor (BFA, *n* = 5; LG, *n* = 6; SOL, *n* = 6; and VL, *n* = 5) muscles in the hindlimbs and one flexor (BB, *n* = 6) and two extensor (ECU, *n* = 5 and TRI, *n* = 7) muscles in the forelimbs. We measured EMG burst duration from onset to offset and mean EMG amplitude by integrating the full-wave rectified EMG burst from onset to offset divided by burst duration. We then normalized EMG amplitudes to the mean control amplitude (i.e., mean amplitude measured during the cycle preceding stimulation) and expressed as a percentage. We performed statistical analysis of the EMG amplitude on normalized data. The same experimenter (A.N.M.) determined burst onsets and offsets of selected muscles by visual inspection from the raw EMG waveforms using a custom-made program.

### Statistics

To evaluate the effects of SP nerve stimulation on spatiotemporal variables and EMG bursts, we performed a nonparametric one-factor [step cycles (control, stimulated, first cycle after stimulation)] Friedman test in each phase. The normality of each variable was assessed by the Shapiro Wilk test. When a main effect was found, a *post hoc* analysis was conducted using Bonferroni’s test. The critical level for statistical significance was set at an α-level of 0.05. Analyses were done with Statistica 8 (Statsoft). All values in the figures are the mean ± SD.

## Results

To determine the effects of perturbations by hindlimb cutaneous inputs on the quadrupedal pattern at different times in the step cycle, we electrically stimulated the SP nerve in four phases and characterized adjustments in the four limbs. We measured and compared kinematic and EMG variables during mid-stance, the stance-to-swing transition, mid-swing, and the swing-to-stance transition of the ipsilateral limb (the limb stimulated). We analyzed variables in a stepping sequence of three successive cycles: the control cycle (cycle before the stimulation, in white), in the stimulated cycle (red), and in the first (dark gray) poststimulation cycle.

### Hindlimb cutaneous inputs produce phase-dependent kinematic and EMG adjustments

**Figure 2.**
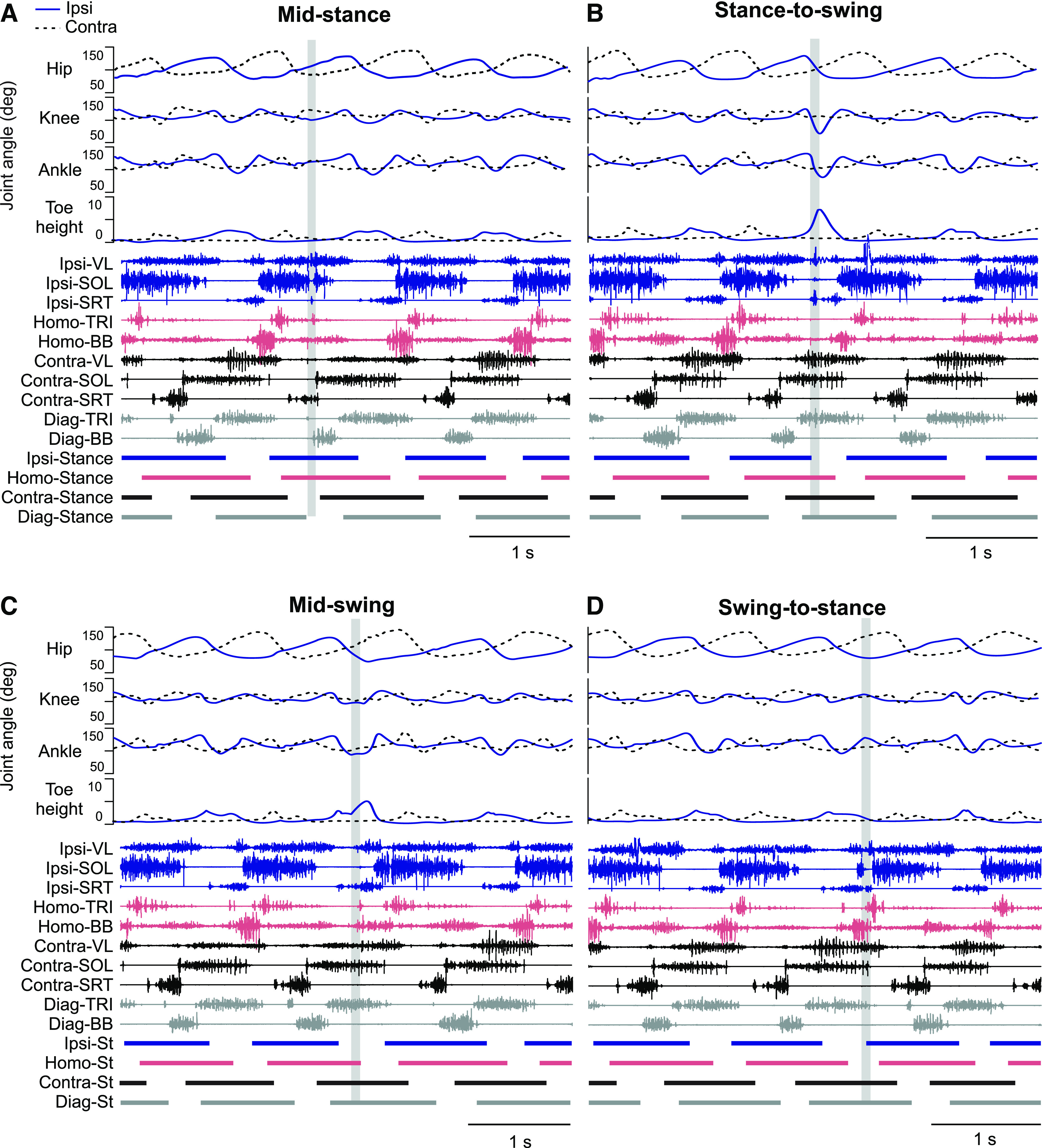
Phase-dependent kinematic and EMG adjustments during locomotion in a single cat. The figure shows joint angles of the ipsilateral and contralateral hindlimb, toe height, EMG from selected muscles along with stance phases of the four limbs with stimulation at mid-stance (***A***), stance-to-swing transition (***B***), mid-swing (***C***), and swing-to-stance transition (***D***). For abbreviations, see Figure 1 legend.

Figure 2 shows the effects of SP nerve stimulation during four phases for a single cat during treadmill locomotion at 0.4 m/s. Each panel shows, from top to bottom, joint angles and toe height of the hindlimbs, the EMG of selected muscles and the stance phases of all four limbs. The vertical shaded area represents the period of SP nerve stimulation. As can be seen, the effects of nerve stimulation were highly dependent on phase. For instance, at mid-stance (Fig. 2*A*) and at the swing-to-stance transition (Fig. 2*D*), we observed a brief inhibition of the ipsilateral SOL EMG activity and brief bursts or increased activity in the ipsilateral SRT and homolateral TRI. In contrast, stimulation at the stance-to-swing transition increased flexion at the three joints of the ipsilateral hindlimb, particularly at the knee, and considerably elevated toe height (Fig. 2*B*). Stimulation at mid-swing prolonged and increased flexion at the knee and ankle and increased toe height as it was starting to decrease (Fig. 2*C*). With stimulation at the stance-to-swing transition or mid-swing, we can observe some EMG changes, such as a brief burst in the ipsilateral VL and SRT. Thus, there are clear differences in the responses evoked depending on where within the step cycle the stimulus is delivered.

### Temporal adjustments of all four limbs with hindlimb cutaneous inputs

**Figure 3.**
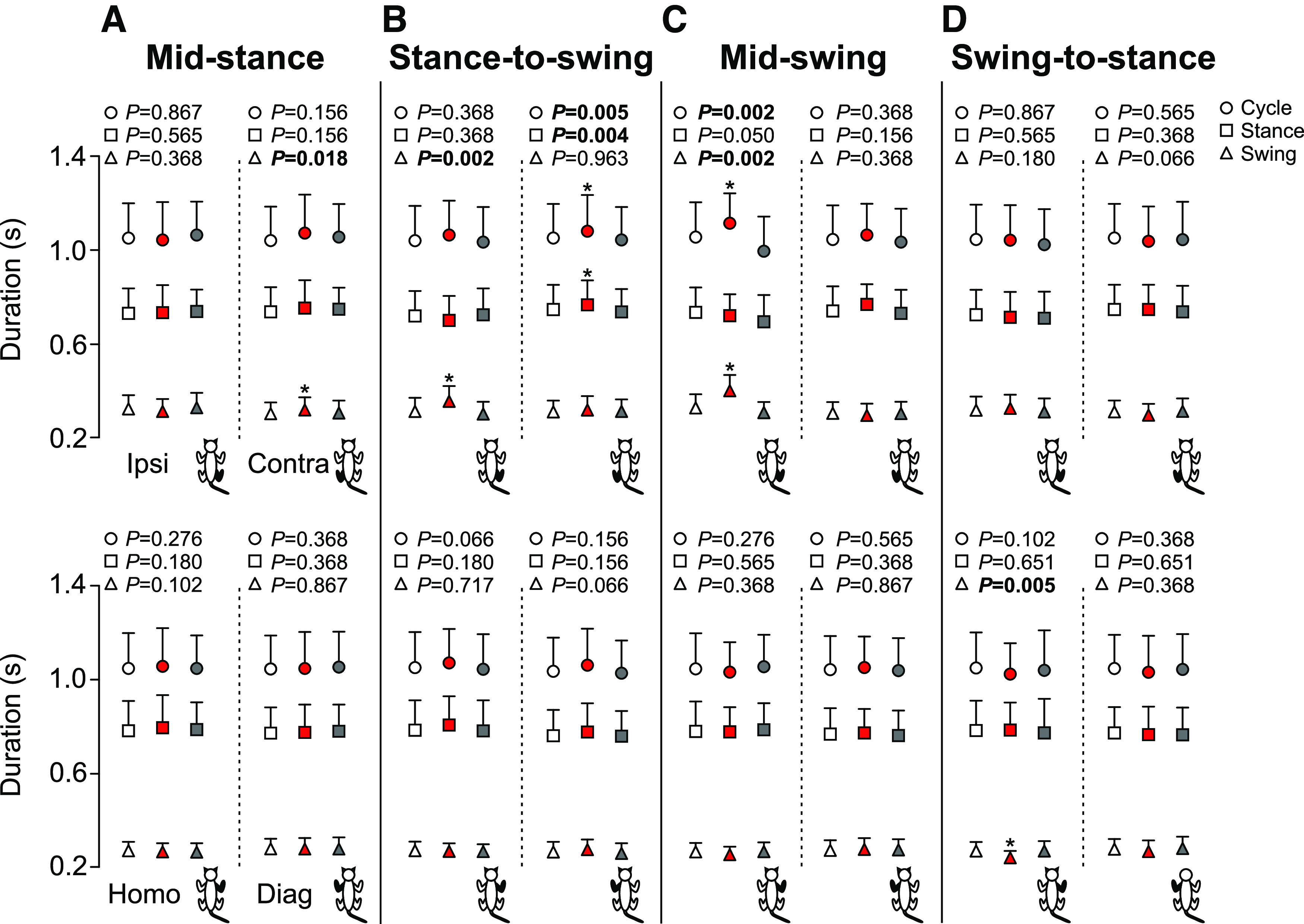
Cycle and phase durations of all four limbs during locomotion across cats. The figure shows cycle, stance and swing durations with stimulation at mid-stance (***A***), stance-to-swing transition (***B***), mid-swing (***C***), and swing-to-stance transition (***D***) in the ipsilateral (Ipsi), contralateral (Contra), homolateral (Homo), and diagonal (Diag) limbs. Control, stimulated and first cycle after stimulation are shown in white, red, and gray, respectively. Each data point represents the mean ± SD for the group (*n* = 7 cats). *p* values comparing the cycles are indicated (main effect of Friedman test) and those in bold highlight significant main effects. The asterisks (*) indicate a significant difference with the control cycle obtained with Bonferroni’s *post hoc* test.

To determine how stimulating the SP nerve affected temporal characteristics of the locomotor pattern, we measured cycle, stance and swing durations in the four limbs (Fig. 3). Note that the right hindlimb is the limb stimulated (ipsilateral). Stimulation during mid-stance had no effect on cycle and phase durations of the ipsilateral hindlimb but significantly increased contralateral swing duration in the stimulated cycle (+5.1%, *p* = 0.018 vs control; Fig. 3*A*). We found no changes in the homolateral and diagonal forelimbs. Stimulation at the stance-to-swing transition significantly increased ipsilateral swing duration in the stimulated cycle (+13.7%, *p* = 002 vs control; Fig. 3*B*) and contralateral cycle and stance duration in the stimulated limb (+2.6%, *p* = 0.005 and +2.8%, *p* = 0.004 vs control, respectively; Fig. 3*B*). We found no changes in the homolateral and diagonal forelimbs. With stimulation at mid-swing, ipsilateral cycle and swing durations were significantly longer in the stimulated cycle (+6.0%, *p* = 0.002 and +23.5%, *p* = 0.002 vs control, respectively; Fig. 3*C*) whereas no changes in cycle and phase durations were found in the three other limbs. Stimulation performed at the swing-to-stance transition only significantly decreased homolateral forelimb swing duration in the stimulated cycle (−10.5%, *p* = 0.005 vs control; Fig. 3*D*).

**Figure 4.**
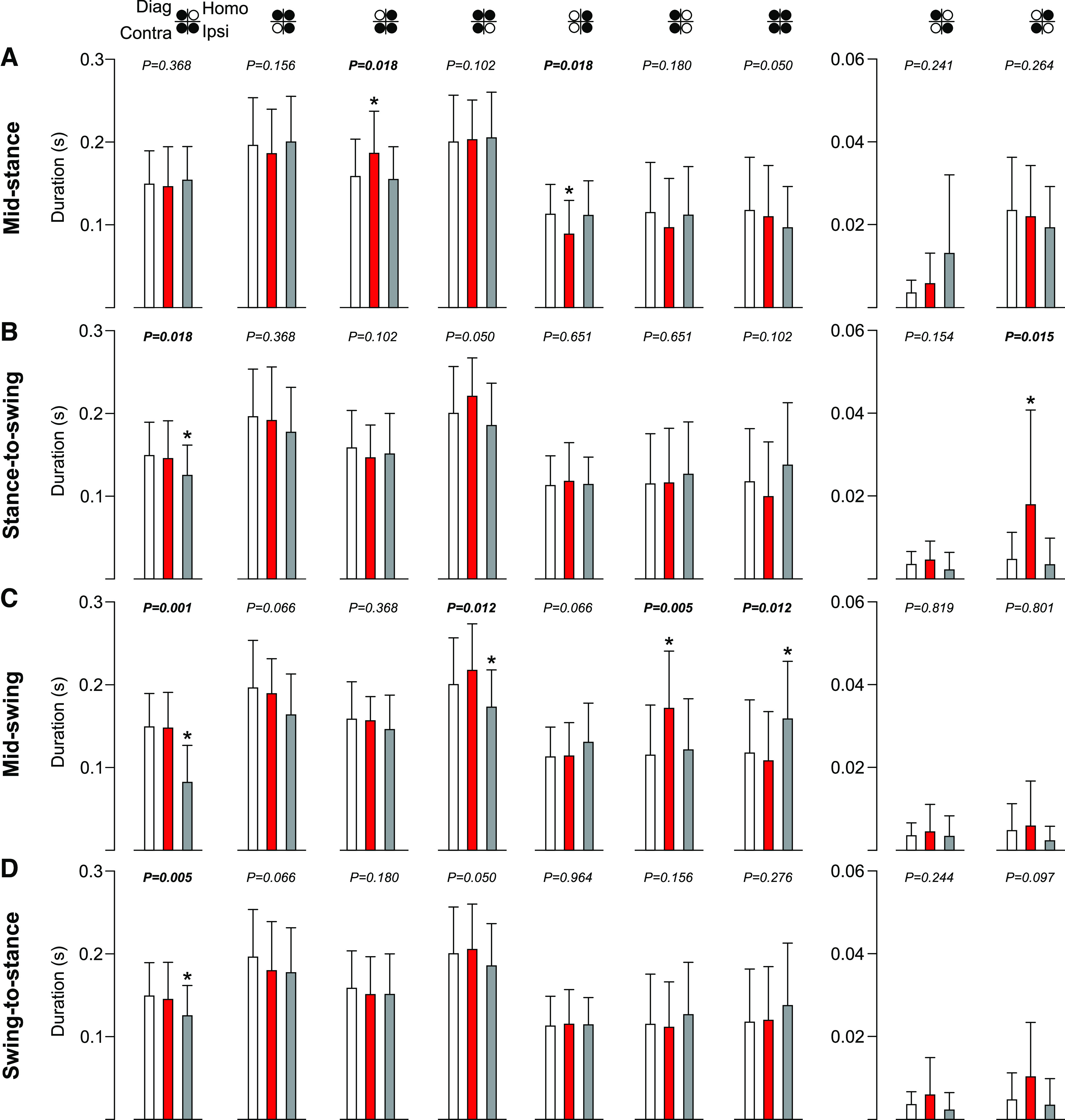
Support periods during locomotion across cats. The figure shows support periods with stimulation at mid-stance (***A***), stance-to-swing transition (***B***), mid-swing (***C***), and swing-to-stance transition (***D***). Control, stimulated and first cycle after stimulation are shown in white, red, and gray, respectively. Each data bar represents the mean ± SD for the group (*n* = 7 cats). *p* values comparing three consecutive cycles are indicated (main effect of Friedman test) and those in bold highlight significant main effects. The asterisks (*) indicate a significant difference with the control cycle obtained with Bonferroni’s *post hoc* test. Ipsi, ipsilateral; Homo, homolateral; Contra, contralateral; Diag, diagonal.

We generally find eight individual support periods during quadrupedal locomotion in a cycle (D’Angelo et al., 2014; Frigon et al., 2014, 2021). Note that a diagonal support period can be replaced by a period of quadrupedal support in some cycles, thus we can find nine different support periods. Figure 4 shows the duration of the support periods in the four stimulation phases. Stimulation at mid-stance increased the triple support period involving the ipsilateral, contralateral and homolateral limbs (+17.7%, *p* = 0.018 vs control) and decreased the ipsilateral homolateral double support periods (−21.4%, *p* = 0.018 vs control; Fig. 4*A*). Stimulation at the stance-to-swing transition increased the diagonal support involving the contralateral and homolateral limbs in the stimulated cycle (+279.2%, *p* = 0.015 vs control), whereas the triple support involving the ipsilateral, contralateral and diagonal limbs decreased in the first cycle after stimulation (−16.0%, *p* = 0.018 vs control; Fig. 4*B*). Stimulation at mid-swing increased the contralateral homolateral double support period in the stimulated cycle (+49.3%, *p* = 0.005 vs control; Fig. 4*C*). We observed a decrease in the duration of two triple support periods in the first cycle after stimulation: ipsilateral, contralateral, and diagonal limbs (−45.0%, *p* = 0.001 vs control) and contralateral, homolateral and diagonal limbs (−13.6%, *p* = 0.012 vs control; Fig. 4*C*). In contrast, the quadrupedal increased (+35.1%, *p* = 0.012 vs control; Fig. 4*C*). At the swing-to-stance transition, we only observed a decrease in the triple support period involving the ipsilateral, contralateral and diagonal limbs during the first cycle after stimulation (−16.0%, *p* = 0.005 vs control; Fig. 4*D*).

### Spatial and kinematic adjustments of all four limbs with hindlimb cutaneous inputs

**Figure 5.**
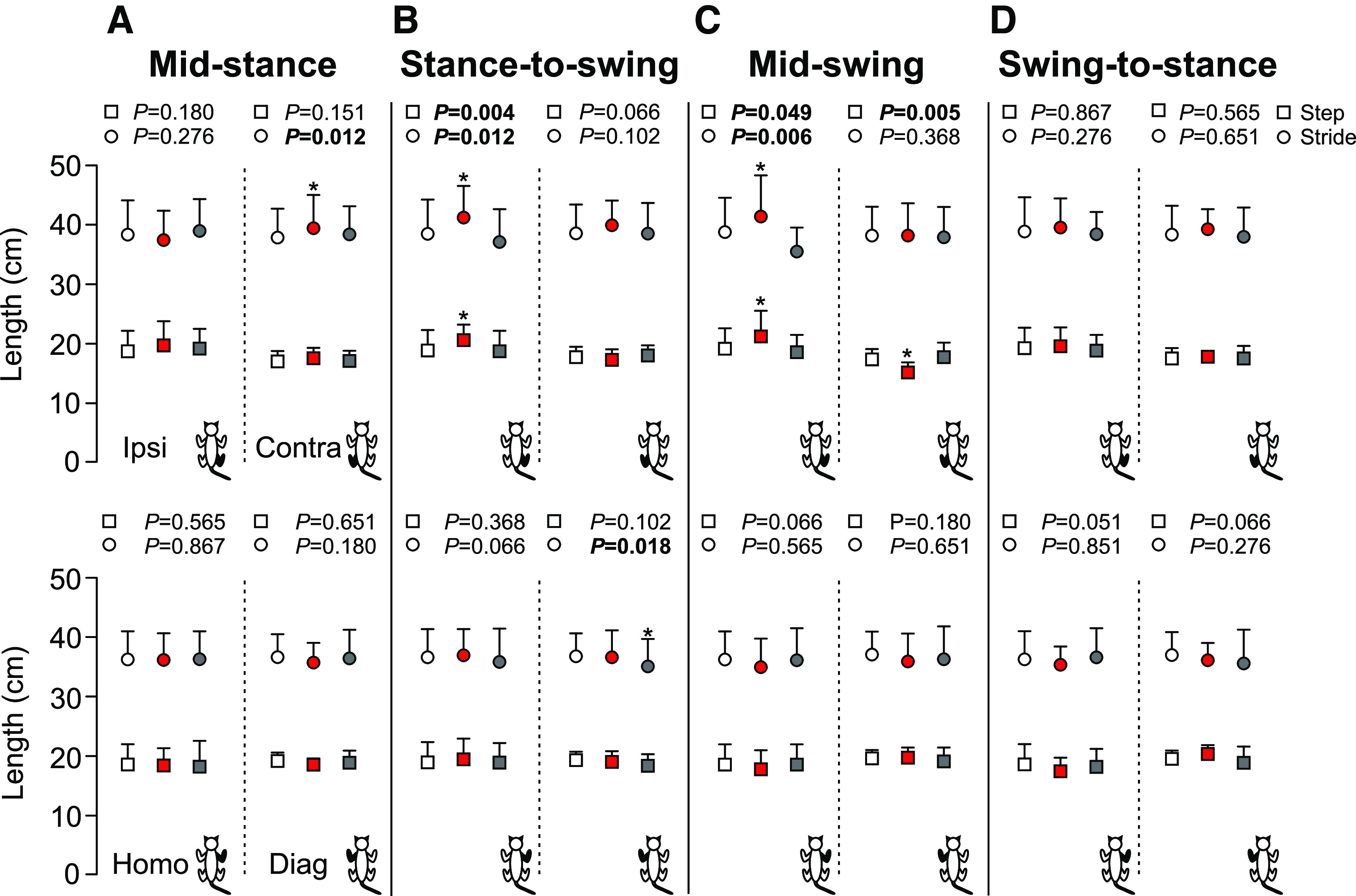
Step and stride lengths of all four limbs during locomotion across cats. The figure shows step and stride lengths with stimulation at mid-stance (***A***), stance-to-swing transition (***B***), mid-swing (***C***), and swing-to-stance transition (***D***) in the ipsilateral (Ipsi), contralateral (Contra), homolateral (Homo), and diagonal (Diag) limbs. Control, stimulated and first cycle after stimulation are shown in white, red, and gray, respectively. Each data point indicates the mean ± SD for the group (*n* = 7 cats). *p* values comparing three consecutive cycles are indicated (main effect of Friedman test) and those in bold highlight significant main effects. The asterisks (*) indicate a significant difference with the control cycle obtained with Bonferroni’s *post hoc* test.

We measured step length to determine the distance between homologous limbs at contact of the leading limb and stride length to determine the distance traveled by all four limbs with stimulation of the SP nerve in the four phases (Fig. 5). Stimulation at mid-stance only significantly increased contralateral stride length in the stimulated cycle (+9,1%, *p* = 0.012 vs control; Fig. 5*A*). With stimulation at the stance-to-swing transition, ipsilateral step and stride lengths were significantly longer in the stimulated cycle (+7.6%, *p* = 0.004 and +6.8%, *p* = 0.012 vs control, respectively; Fig. 5*B*) and diagonal stride length was significantly shorter in the first cycle after stimulation (−5.4%, *p* = 0.018 vs control; Fig. 5*B*). Step and stride lengths of contralateral and homolateral limbs were unaffected. With stimulation at mid-swing, ipsilateral step and stride lengths were significantly longer in the stimulated cycle (+9.1%, *p* = 0.049 and +5.7%, *p* = 0.006 vs control, respectively; Fig. 5*C*), whereas the contralateral step length was shorter (−13.3%, *p* = 0.005 vs control; Fig. 5*C*). Stimulation at the swing-to-stance transition did not affect step and stride lengths of any of the four limbs. Thus, with stimulation at the stance-to-swing transition and at mid-swing, the ipsilateral hindlimb travels a greater distance.

#### Heights and angles

**Figure 6.**
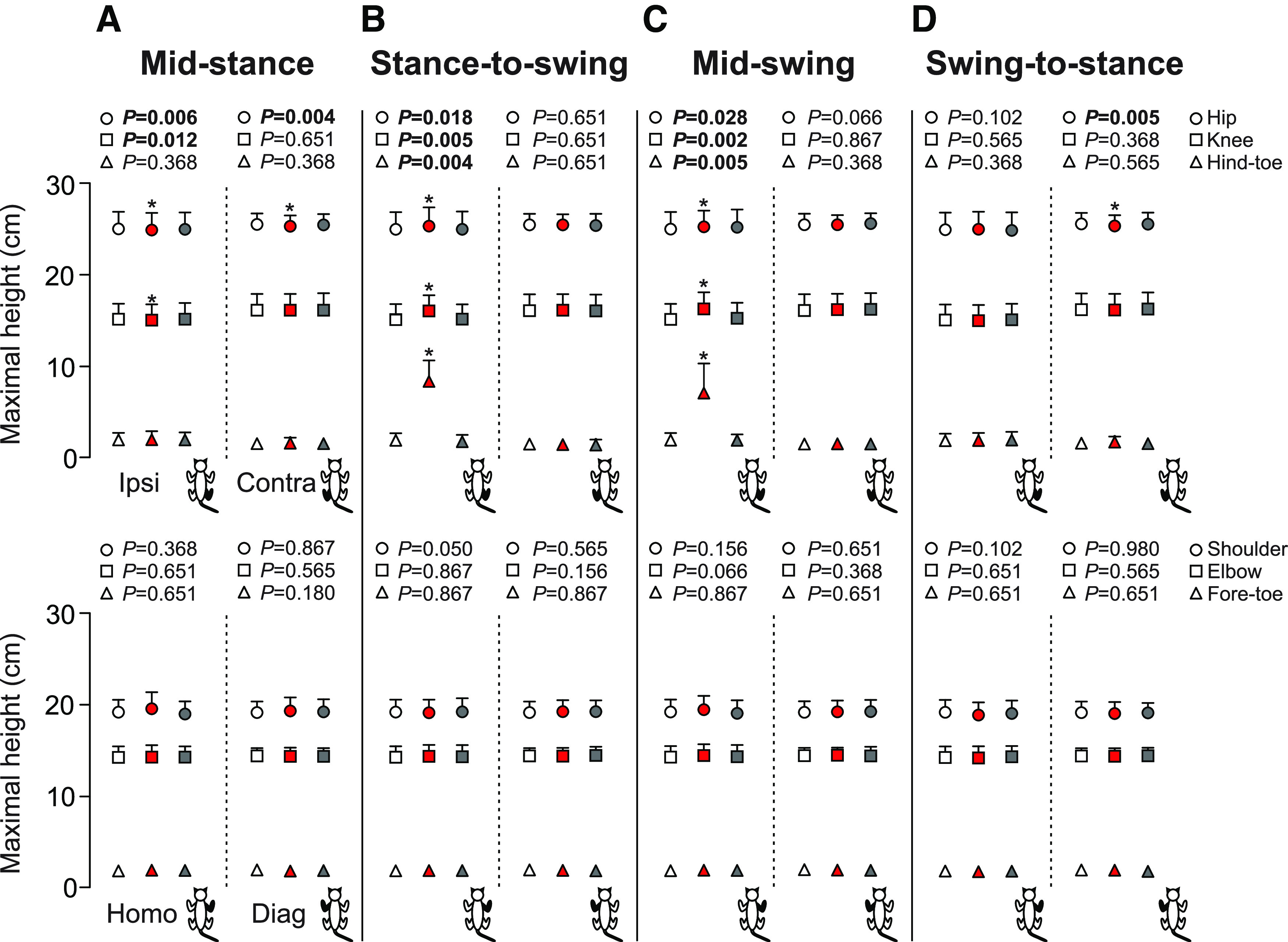
Joint and toe heights in the four limbs during locomotion across cats. The figure shows maximum heights of the hip, knee, hind-toe, shoulder, elbow, and fore-toe with stimulation at mid-stance (***A***), stance-to-swing transition (***B***), mid-swing (***C***), and swing-to-stance transition (***D***). Control, stimulated and first cycle after stimulation are shown in white, red, and gray, respectively. Each data point represents the mean ± SD for the group (*n* = 7 cats). *p* values comparing the cycles are indicated (main effect of Friedman test) and those in bold highlight significant main effects. The asterisks (*) indicate a significant difference with the control cycle obtained with Bonferroni’s *post hoc* test. Ipsi, ipsilateral; Homo, homolateral; Contra, contralateral; Diag, diagonal.

Hindlimb cutaneous inputs alter the maximum height of the hip, knee and toes in the hindlimbs, and the shoulder, elbow and toes in the forelimbs, in a phase-dependent manner (Fig. 6). Overall, homolateral and diagonal shoulder, elbow and forelimb toe heights were not altered by SP nerve stimulation in all locomotor phases (Fig. 6). In the hindlimbs, stimulation performed at mid-stance significantly lowered the ipsilateral and contralateral hip in the stimulated cycle (−0.8%, *p* = 0.006 and −0.9%, *p* = 0.004 vs control, respectively; Fig. 6*A*). Ipsilateral knee height was also significantly lowered in the stimulated cycle (*p* = 0.012 vs control; Fig. 6*A*). We found no change for the ipsilateral and contralateral toe heights and contralateral knee height. With stimulation at the stance-to-swing transition, ipsilateral hip, knee and toe heights were significantly elevated in the stimulated cycle (+1.5%, *p* = 0.018; +6.1%, *p* = 0.005; +358.4%, *p* = 0.004 vs control, respectively; Fig. 6*B*), whereas the contralateral hip, knee and toe heights were unaffected. Stimulation performed at mid-swing significantly elevated ipsilateral hip, knee and toe heights in the stimulated cycle (+1.5%, *p* = 0.028; +7.5%, *p* = 0.002 and +231.3%, *p* = 0.005 vs control, respectively; Fig. 6*C*). With stimulation at the swing-to-stance transition, only the contralateral hip was lowered in the stimulated cycle (−0.9%, *p* = 0.005 vs control; Fig. 6*D*).

**Figure 7.**
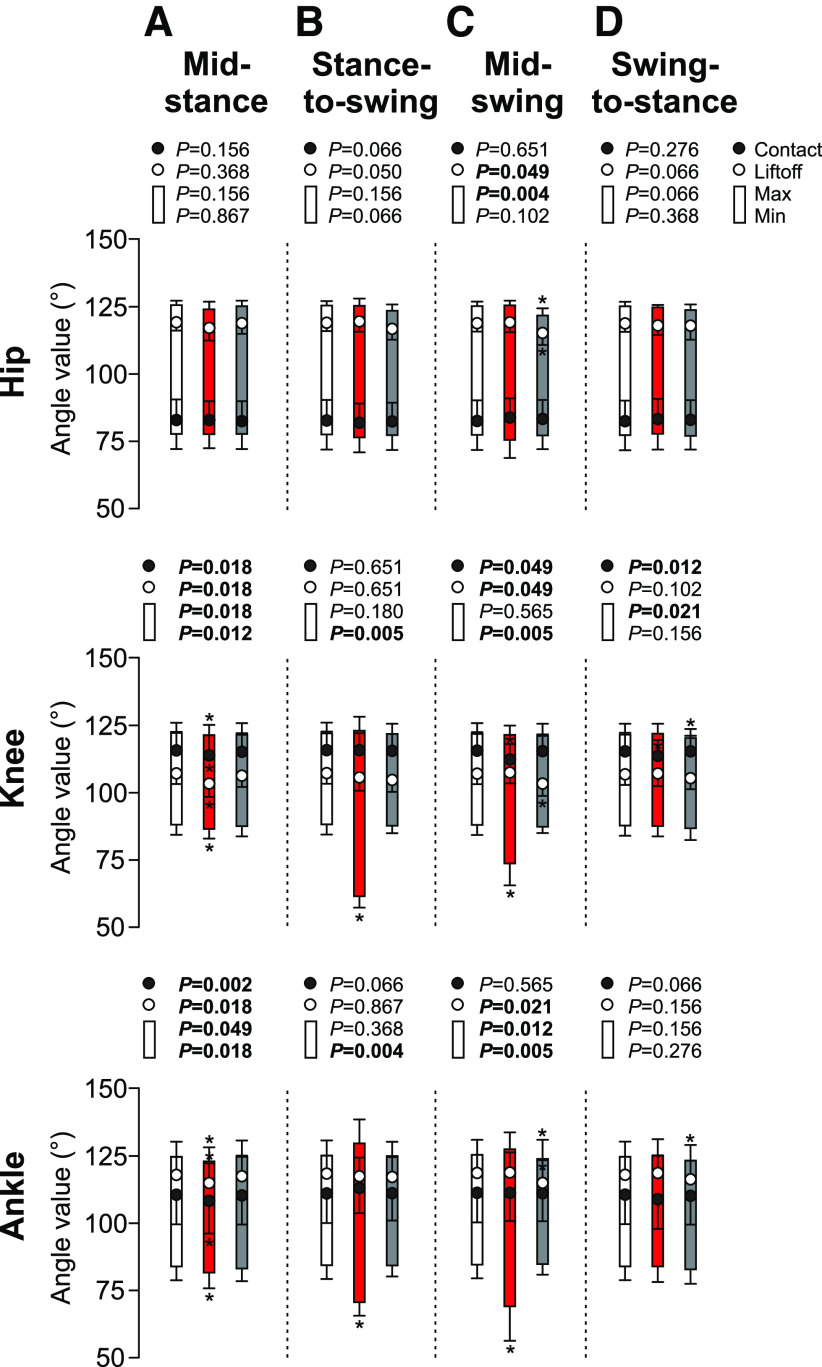
Joint angles of the ipsilateral limb during locomotion across cats. The figure shows hip, knee and ankle joint angles of the ipsilateral limb at contact, liftoff as well as the maximum and minimum values with stimulation at mid-stance (***A***), stance-to-swing transition (***B***), mid-swing (***C***), and swing-to-stance transition (***D***). Control, stimulated and first cycle after stimulation are shown in white, red, and gray, respectively. *p* values comparing three consecutive cycles are indicated (main effect of Friedman test) and those in bold highlight significant main effects. The asterisks (*) indicate a significant difference with the control cycle obtained with Bonferroni’s *post hoc* test.

Cutaneous inputs from the SP nerve also generated phase-dependent changes in joint angles in the ipsilateral hindlimb (Fig. 7). With stimulation at mid-stance, ipsilateral knee and ankle joints had smaller maximum angles in the stimulated cycle (−1.3%, *p* = 0.018 and −1.7%, *p* = 0.049 vs control, respectively; Fig. 7*A*). Knee and ankle joints also had significantly smaller minimum angles (i.e., greater flexion) in the stimulated cycle (−2.0%, *p* = 0.012 and −2.7%, *p* = 0.018 respectively; Fig. 7*A*). At contact, knee and ankle joints were significantly smaller (i.e., more flexed) in the stimulated cycle (−2.2%, *p* = 0.018 and −2.7%, *p* = 0.002 vs control, respectively; Fig. 7*A*). The same observations were made at liftoff (−2.9%, *p* = 0.018 and −2.6%, *p* = 0.018 vs control, respectively; Fig. 7*A*). We found no change for hip angle. With stimulation at the stance-to-swing transition, ipsilateral knee and ankle joints had smaller minimum angles (i.e., greater flexion) in the stimulated cycle (−29.2%, *p* = 0.005 and −15.2%, *p* = 0.004 vs control, respectively; Fig. 7*B*), with no change in hip angle. At contact and liftoff, ipsilateral hindlimb joints were unaffected. With stimulation at mid-swing, knee and ankle joints had significantly smaller minimum angles (i.e., greater flexion) in the stimulated cycle (−16.1%, *p* = 0.005 and −18.8%, *p* = 0.005 vs control, respectively; Fig. 7*C*). The hip and ankle joints also had smaller maximum angles (−3.2%, *p* = 0.004 and −1.3%, *p* = 0.012 respectively; Fig. 7*C*) in the first cycle after stimulation. At liftoff, the hip, knee and ankle joints at liftoff were significantly more flexed in the first cycle after stimulation (−3.7%, *p* = 0.049; −4.2%, *p* = 0.049; and −3.0%, *p* = 0.021 vs control, respectively; Fig. 7*C*). At contact, the knee joint had was significantly more flexed in the stimulated cycle (−0.3%, *p* = 0.049 vs control; Fig. 7*C*) whereas no change was reported in hip and ankle joint angles. Stimulation at the swing-to-stance transition only decreased knee angle at contact in the stimulated cycle (−1.0%, *p* = 0.012 vs control; Fig. 7*D*). Knee joint also had smaller maximum angles in the first cycle following the stimulation (−0.9%, *p* = 0.021 vs control, respectively; Fig. 7*D*).

### Muscle activity adjustments of all four limbs with hindlimb cutaneous inputs

#### EMG burst duration

**Figure 8.**
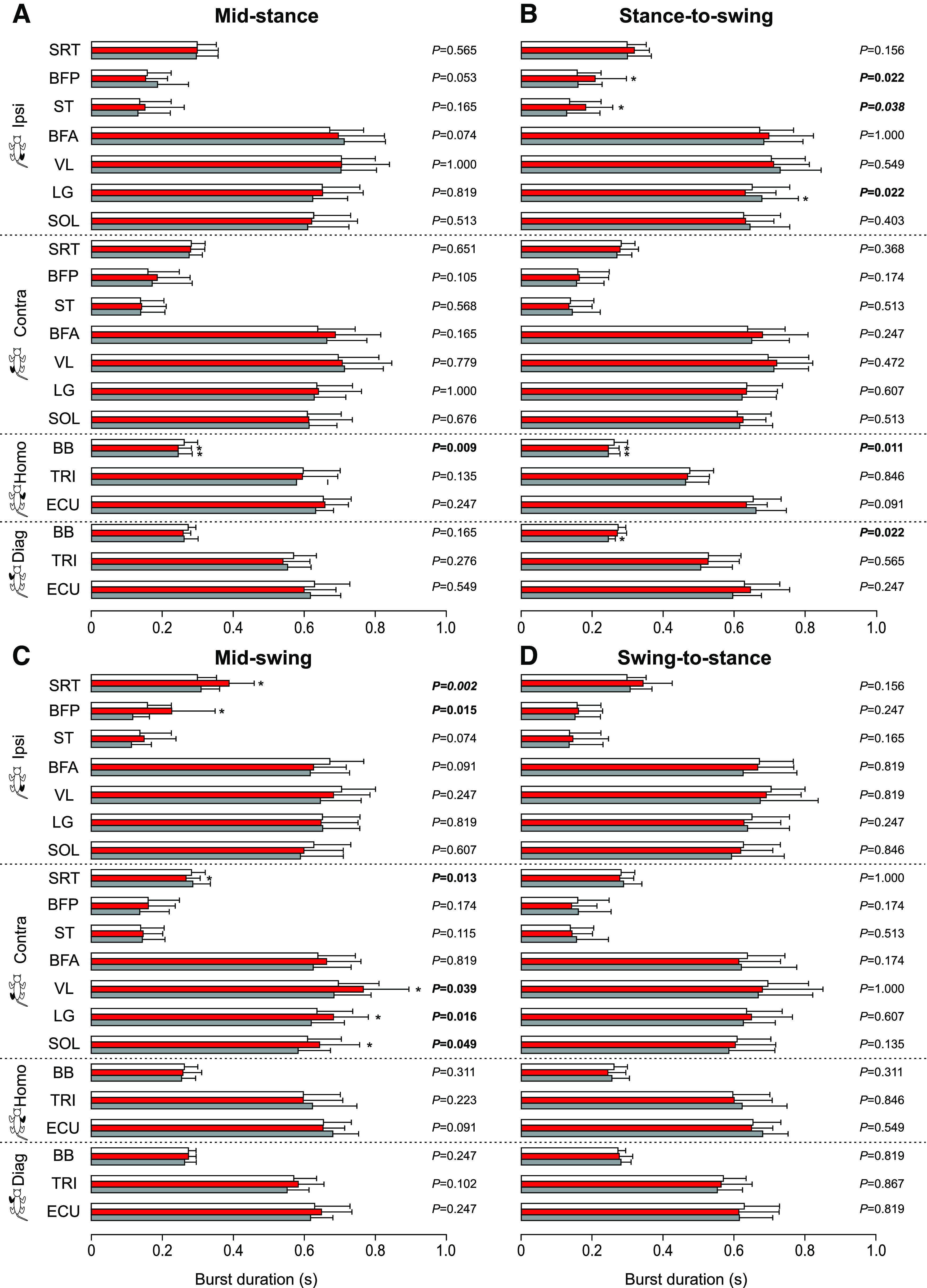
Burst durations in all four limbs during locomotion across cats. The figure shows EMG burst durations in selected muscles with stimulation at mid-stance (***A***), stance-to-swing transition (***B***), mid-swing (***C***), and swing-to-stance transition (***D***) in the ipsilateral (Ipsi), contralateral (Contra), homolateral (Homo), and diagonal (Diag) limbs. Control, stimulated and first cycle after stimulation are shown in white, red, and gray, respectively. SRT, anterior sartorius (*n* = 7 cats); BFP, biceps femoris posterior (*n* = 5 cats); ST, semitendinosus (*n* = 6 cats); BFA, biceps femoris anterior (*n* = 5 cats); VL, vastus lateralis (*n* = 5 cats); LG, lateral gastrocnemius (*n* = 6 cats); SOL, soleus (*n* = 6 cats); BB, biceps brachii (*n* = 6 cats); TRI, the long head of the triceps brachii (*n* = 7 cats); ECU, extensor carpi ulnaris (*n* = 5 cats). *p* values comparing three consecutive cycles are indicated (main effect of Friedman test) and those in bold highlight significant main effects. The asterisks (*) indicate a significant difference with the control cycle obtained with Bonferroni’s *post hoc* test.

For burst duration, we found a significant decrease with stimulation at mid-stance (Fig. 8*A*) for the homolateral BB in the stimulated cycle (−6.6%, *p* = 0.009 vs control) and in the first cycle after the stimulation (−6.5%, *p* = 0.009 vs control). With stimulation at the stance-to-swing transition (Fig. 8*B*), we found a significant increase of EMG burst duration for the ipsilateral BFP (+31.9%, *p* = 0.022 vs control) and ST (+33.1%, *p* = 0.038 vs control) in the stimulated cycle and for the ipsilateral LG (+4.2%, *p* = 0.022 vs control) in the first cycle after stimulation. In contrast, we found a significant decrease of EMG duration for the homolateral BB in the stimulated cycle (−6.2%, *p* = 0.011 vs control) and in the first cycle after the stimulation (−6.2%, *p* = 0.011 vs control), and for the diagonal BB (−9.9%, *p* = 0.022 vs control) only in the first cycle after the stimulation. Most changes in EMG burst duration occurred with stimulation at mid-swing (Fig. 8*C*). We observed a significant increase of EMG burst duration for the ipsilateral SRT (+29.9%, *p* = 0.002 vs control) and BFP (+43.3%, *p* = 0.015 vs control) and the contralateral VL (+10.1%, *p* = 0.039 vs control), LG (+7.3%, *p* = 0.016 vs control) and SOL (+5.6%, *p* = 0.049 vs control) in the stimulated cycle. In contrast, we observed a significant decrease of EMG duration for the contralateral SRT (−5.5%, *p* = 0.013 vs control) in the stimulated cycle. With stimulation at the swing-to-stance transition (Fig. 8*D*), no significant changes were found.

#### EMG amplitude

**Figure 9.**
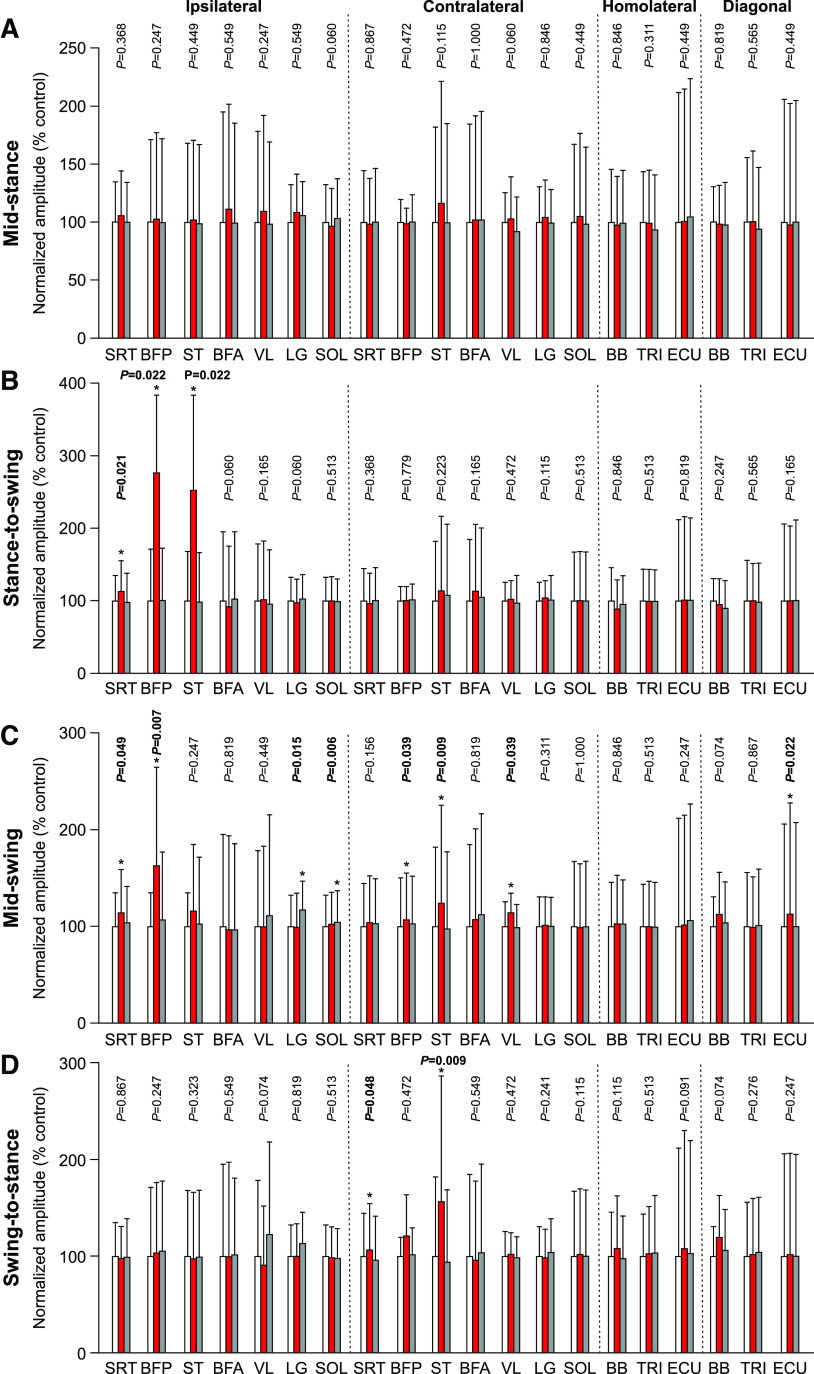
Burst amplitudes in all four limbs during locomotion across cats. The figure shows EMG burst amplitudes in selected muscles with stimulation at mid-stance (***A***), stance-to-swing transition (***B***), mid-swing (***C***), and swing-to-stance transition (***D***) in the ipsilateral (Ipsi), contralateral (Contra), homolateral (Homo), and diagonal (Diag) limbs. Control, stimulated and first cycle after stimulation are shown in white, red, and gray, respectively. SRT, anterior sartorius (*n* = 7 cats); BFP, biceps femoris posterior (*n* = 5 cats); ST, semitendinosus (*n* = 6 cats); BFA, biceps femoris anterior (*n* = 5 cats); VL, vastus lateralis (*n* = 5 cats); LG, lateral gastrocnemius (*n* = 6 cats); SOL, soleus (*n* = 6 cats); BB, biceps brachii (*n* = 6 cats); TRI, the long head of the triceps brachii (*n* = 7 cats); ECU, extensor carpi ulnaris (*n* = 5 cats). *p* values comparing three consecutive cycles are indicated (main effect of Friedman test) and those in bold highlight significant main effects. The asterisks (*) indicate a significant difference with the control cycle obtained with Bonferroni’s *post hoc* test.

For mean EMG amplitude, no significant changes were found with stimulation at mid-stance (Fig. 9*A*). With stimulation at the stance-to-swing transition (Fig. 9*B*), we observed a significant increase of EMG amplitude for the three flexor muscles of the hindlimbs (SRT, BFP, and ST) in the stimulated cycle. The two largest increases occurred for the ipsilateral BFP (+243.4%, *p* = 0.022 vs control) and ST (+251.0%, *p* = 0.022 vs control), with a smaller increase for the ipsilateral SRT (+13.0%, *p* = 0.021 vs control). As with burst duration, most significant changes in EMG amplitude occurred with stimulation at mid-swing (Fig. 9*C*). In the hindlimbs, we found a significant increase of EMG amplitude for the ipsilateral/contralateral BFP (+78.3%, *p* = 0.007 and +20.8%, *p* = 0.039 vs control, respectively), the contralateral ST (+31.4%, *p* = 0.009 vs control) and VL (+15.5%, *p* = 0.039 vs control) and the ipsilateral SRT (+13.6%, *p* = 0.049 vs control) in the stimulated cycle. We found a significant increase for the ipsilateral LG (+20.0%, *p* = 0.015 vs control) and SOL (+4.8%, *p* = 0.006 vs control) in the first cycle after stimulation. In the forelimbs, we only observed a significant increase for the diagonal ECU (+16.4%, *p* = 0.022 vs control) in the stimulated cycle. With stimulation at the swing-to-stance transition (Fig. 9*D*), we observed a significant increase of EMG amplitude for the contralateral SRT (+7.0%, *p* = 0.048 vs control) and ST (+53.3%, *p* = 0.009 vs control) in the stimulated cycle.

## Discussion

The main purpose of this study was to determine how the quadrupedal locomotor pattern adjusts to a sensory perturbation evoked by electrically stimulating cutaneous afferents of the SP nerve with a relatively long train at four different phases of the step cycle. Studies to date have focused almost exclusively on adjustments in the ipsilateral and contralateral hindlimbs/legs in cats and humans (Forssberg, 1979; Schillings et al., 1996; Lam et al., 2003; Quevedo et al., 2005a,b). Below, we discuss how stimulating the SP nerve led to phase-dependent kinematic and EMG adjustments in the four limbs during quadrupedal locomotion in intact cats.

### Functional effects of the sensory perturbation on the locomotor pattern are phase dependent

The SP nerve innervates the skin of the foot dorsum (Bernard et al., 2007). Thus, electrically stimulating SP afferents simulates a contact to the foot dorsum. Mechanical or electrical stimulation of the foot dorsum or the SP nerve has been used to describe the stumbling corrective reaction in cats and humans (Forssberg, 1979; Wand et al., 1980; Buford and Smith, 1993; Schillings et al., 1996; Zehr et al., 1997; Quevedo et al., 2005a,b; Hurteau et al., 2018). We recently showed that stimulating the SP nerve during treadmill locomotion evoked phase-dependent reflex responses in all four limbs in intact cats (Hurteau et al., 2018). In that study, however, we delivered short trains (three pulses) just above the motor threshold to evoke reflex responses without noticeably perturbing the pattern. In other words, we wanted to avoid a large kinematic change in the gait pattern to focus on cutaneous reflex responses. In the present study, we used longer trains (25 pulses), to generate a large functional response during mid-swing, consisting mainly of pronounced knee flexion. At this stimulation intensity, it is unlikely that we activated high threshold nociceptive afferents that mediate the withdrawal reflex because such activation elicits flexion withdrawal throughout the locomotor cycle, including during the stance phase as well as during quiet standing (Forssberg, 1979; Buford and Smith, 1993; Zehr et al., 1997). Moreover, cats did not display discomfort with stimulation. Thus, it is likely that temporal summation of inputs from low-threshold SP afferents evoked the large response observed with stimulation at mid-swing and at the stance-to-swing transition. We used the same stimulation intensity to determine the functional effects of activating SP nerve afferent inputs in all four limbs at four phases: mid-stance, the stance-to-swing transition, mid-swing, and the swing-to-stance transition. These four phases have different functional requirements to maintain balance and ensure forward progression when faced with a sensory perturbation to the foot dorsum. We will first discuss the effects of stimulation at mid-swing and the stance-to-swing transition followed by stimulation at mid-stance and at the swing-to-stance transition.

Not surprisingly, we observed the largest functional effects of the stimulation at mid-swing and at the stance-to-swing transition, as observed by other studies in various cat preparations (Forssberg, 1979; LaBella et al., 1992; Quevedo et al., 2005a,b; Hurteau et al., 2018) and during human locomotion (Schillings et al., 1996; Zehr et al., 1997; Lam et al., 2003). At mid-swing, the stimulated hindlimb is off the ground and the other three limbs must ensure balance to allow the stimulated hindlimb to alter its trajectory. At the stance-to-swing transition, swing is just beginning and the stimulated limb can prolong either its stance or produce a stumbling corrective reaction. With stimulation at mid-swing and the stance-to-swing transition, the knee and ankle of the ipsilateral hindlimb flexed significantly more compared with the control cycle (Fig. 7). We also observed an increase in hip flexion with stimulation at mid-swing. Flexion of the joints considerably increased toe height (Fig. 6). Increased activity of BFP and/or ST (knee flexors/hip extensors) as well as SRT anterior (hip flexor/knee extensor), along with an earlier onset or later offset of their activity were responsible for increased knee and hip flexion (Figs. 8, 9). Increased and prolonged joint flexion and flexor activity increased swing duration (Fig. 3), producing longer stride and step lengths (Fig. 5). Our results are consistent with several studies that described increased activity in knee, ankle, and hip flexors with electrical stimulation of the foot dorsum during swing in intact and chronic spinal cats or in the flexor phase during fictive locomotion in decerebrate curarized cats (Forssberg et al., 1977; Forssberg, 1979; Quevedo et al., 2005a,b). The stumbling corrective reaction in humans evoked mechanically or with electrical stimulation of the SP nerve shares similar characteristics with the cat (Schillings et al., 1996; Zehr et al., 1997; Haridas and Zehr, 2003). In both cats and humans, increased activity in ankle extensors and/or suppression of ankle flexors precedes increased activity in ankle flexors during the stumbling corrective reaction with mechanical and/or electrical stimulation of the foot dorsum during swing (Buford and Smith, 1993; Van Wezel et al., 1997; Zehr et al., 1997; Haridas and Zehr, 2003; Quevedo et al., 2005a,b). Coupled with knee flexion, this helps move the foot up and away from the contact before the hip and ankle flex to move the limb forward and over the obstacle. Ankle extension/plantarflexion is sometimes absent with electrical stimulation, with only ankle and knee flexion observed (Buford and Smith, 1993).

What adjustments do we observe in the other three limbs with stimulation at mid-swing and at the stance-to-swing transition? We observed an increase in cycle and stance phase durations of the contralateral hindlimb with stimulation at the stance-to-swing transition in the stimulated cycle, but not with stimulation at mid-swing (Fig. 3). Burst durations of contralateral extensors, such as VL, SOL, and LG, increased during the stimulated cycle with stimulation at mid-swing (Fig. 8). We also observed an increase in contralateral VL amplitude with stimulation at mid-swing (Fig. 9). A few studies have reported increased activity and/or excitatory reflex responses in contralateral extensors in intact cats with electrical stimulation of the foot dorsum during swing (Forssberg, 1979; Duysens and Loeb, 1980). A short-latency crossed inhibitory pathway from the SP nerve to ankle extensors is also present during locomotion in intact cats and could help prolong the contralateral stance phase when the ipsilateral hindlimb is perturbed during swing (Frigon and Rossignol, 2008a). With stimulation at mid-swing and at the stance-to-swing transition, we observed no changes in swing durations of the contralateral hindlimb or in phase durations of the homolateral and diagonal forelimbs. Measuring changes in support periods revealed how cats adjusted their interlimb pattern to the perturbation (Fig. 4). With stimulation at the stance-to-swing transition, the diagonal period involving the contralateral hindlimb and homolateral forelimb increased during the stimulated cycle while the triple support period involving both hindlimbs and the diagonal forelimb decreased in the first cycle after stimulation. With stimulation at mid-swing, the contralateral homolateral support period increased during the stimulated cycle, thus shifting support to the contralateral side, followed by a decrease in the first cycle after stimulation in two support periods and an increase in quad support. Therefore, adjustments in the interlimb pattern contributed to dynamic balance in response to a sensory perturbation at mid-swing and the stance-to-swing transition.

Stimulating the SP nerve at mid-stance or at the swing-to-stance transition produced more subtle changes in the pattern. With a perturbation in these phases, the goal is not to correct the trajectory of the stimulated limb but to prevent stumbling. Stimulation at these two phases did not produce changes in stride and step lengths in the four limbs, with the exception of a small increase in stride length of the contralateral hindlimb with stimulation at mid-stance (Fig. 5). We did observe some slightly greater flexions at the knee and ankle joints in the stimulated cycle at mid-stance along with a decrease in hip height of the ipsilateral and/or contralateral hindlimbs in both phases, as observed by others (Forssberg, 1979; Buford and Smith, 1993). Increased knee and ankle flexion during stance with SP nerve stimulation is due mainly to a period of inhibition in knee and ankle extensors and to a lesser degree to weak excitatory responses in flexor muscles (Forssberg, 1979; Duysens and Loeb, 1980; Buford and Smith, 1993; Quevedo et al., 2005a,b; Frigon and Rossignol, 2008a; Hurteau et al., 2018). With stimulation at mid-stance, we observed an increase in the triple support period involving the two hindlimbs and the homolateral forelimb during the stimulated cycle with a decrease in the ipsilateral homolateral support period. Thus, with a perturbation at mid-stance, stumbling is prevented by reducing double support and increasing triple support. With stimulation at the swing-to-stance transition, the period of triple support involving both hindlimbs and the diagonal forelimb is reduced in the first cycle after stimulation. In other words, a small correction occurs in the cycle after the perturbation.

During human locomotion, stimulating the SP nerve evokes cutaneous reflexes in the four limbs and although it modifies leg kinematics, particularly at the ankle joint, it does not alter arm kinematics or the structure of its cycle when stimuli are given at any point in the cycle (Haridas and Zehr, 2003). Thus, although humans have maintained a quadrupedal-like coordination pattern during locomotion (Dietz and Michel, 2009; Frigon, 2017; Pearcey and Zehr, 2019), the neural linkages from SP afferents to the arms appear weaker than in cats. The lack of functional effects of SP nerve stimulation on arm swing kinematics during human locomotion is not surprising because humans must frequently use their arms during walking independently of the normal rhythmic pattern with the legs (Meyns et al., 2013). In contrast, cats need to stabilize forelimb support when the hindlimb is perturbed to maintain dynamic balance.

#### Mechanisms coordinating the four limbs in response to a sensory perturbation

Interlimb coordination depends on complex dynamic interactions between spinal circuits that generate the basic pattern of locomotion, somatosensory feedback that informs the central nervous system of changes within the body and the environment, and supraspinal structures that regulate posture and volitional aspects of locomotion (Frigon, 2017). However, neural mechanisms are not the only factor coordinating the limbs. Properties of the musculoskeletal system also play an important role. We recently showed that adult cats with a complete spinal transection at low thoracic levels produced stable quadrupedal locomotion over a range of speeds, despite no neural communication between the brain/cervical cord and the lumbar cord (Audet et al., 2022).

The presence of the stumbling corrective reaction with stimulation of the foot dorsum or the SP nerve indicates a spinal origin, at least for controlling adjustments in the hindlimbs (Forssberg, 1979). Low-threshold cutaneous afferents from the foot dorsum or SP nerve activate several spinal pathways that evoke excitatory and inhibitory post-synaptic potentials in hindlimb motoneurons (Forssberg, 1979; Schmidt et al., 1989; Quevedo et al., 2005b). Stimulating the SP nerve can reset the locomotor rhythm during fictive locomotion in decerebrate curarized cats, consistent with direct access to the spinal locomotor central pattern generator (Stecina et al., 2005; Quevedo et al., 2005a). Interlimb reflexes described in cats (Hurteau et al., 2018) and humans (Haridas and Zehr, 2003) occurring at short latencies likely help to rapidly coordinate muscle activity in the four limbs to stabilize the musculoskeletal system. These short-latency interlimb reflexes, with an onset of <20 ms in cats, are likely confined to the spinal cord, involving commissural interneurons at segmental levels and short or long propriospinal pathways coupling the forelimbs and hindlimbs (Frigon, 2017; Frigon et al., 2021). Longer-latency interlimb reflex responses, >25 ms in cats, can involve supraspinal structures, and occur rapidly enough to assist in altering limb trajectory and making postural corrections (Frigon et al., 2021). Despite the involvement of supraspinal structures, we do not think that voluntary corrections played an important part, if at all, in responding to sensory perturbations.

A few mechanisms can mediate the phase-dependent effects of SP nerve stimulation on the locomotor pattern. Phase-dependent modulation of cutaneous reflexes occurs in spinal cats, consistent with a spinal mechanism (Forssberg et al., 1975, 1977; LaBella et al., 1992; Frigon and Rossignol, 2008a; Hurteau et al., 2017; Hurteau and Frigon, 2018). As an example, increases in burst amplitudes of ipsilateral flexors and contralateral extensors with SP nerve stimulation peaks during swing when these muscles are active (Fig. 9). Thus, the more depolarized state of motoneurons can explain the larger excitatory reflex responses in these muscles. However, studies have shown that modulation of reflex responses is often independent of the background level of EMG, consistent with gating of the transmission in reflex pathways at premotoneuronal levels, which includes modulatory sites in the spinal interneuronal network and primary afferents that project to the spinal cord (Andersson et al., 1978; Duysens and Loeb, 1980; Buford and Smith, 1993; Van Wezel et al., 1997; Zehr et al., 1997, 1998; Haridas and Zehr, 2003; Quevedo et al., 2005a,b; Bui et al., 2013; Hurteau et al., 2017, 2018). Short and longer latency excitatory responses in a given muscle can also be independently modulated during the locomotor cycle. It has been proposed that the spinal locomotor central pattern generator gates transmission in cutaneous reflex pathways because cutaneous reflexes are modulated during fictive locomotion in decerebrate curarized spinal-transected cats (Andersson et al., 1978; LaBella et al., 1992). Indeed, spinal interneurons regulate the release of neurotransmitter from the synaptic terminals of cutaneous afferents, including those from the SP nerve, through presynaptic inhibition/primary afferent depolarization in a phase-dependent manner, as shown during fictive locomotion in decerebrate curarized spinal-intact and spinal-transected cats (Gossard et al., 1989, 1990; Gossard and Rossignol, 1990). Pathways originating in the cervical cord, such as the central pattern generator controlling the forelimbs, and in supraspinal structures, also modulate transmission in hindlimb cutaneous reflex pathways (Engberg et al., 1968; Hongo et al., 1969; Bretzner and Drew, 2005; Sirois et al., 2013). Therefore, several mechanisms can contribute to cutaneous reflex modulation with phase during locomotion.

#### Concluding remarks

In this study, we showed that stimulating cutaneous afferents innervating the foot dorsum generates functional responses involving the whole-body. These responses are phase-dependent and serve to correct or prevent stumbling. We are currently investigating functional responses evoked in the four limbs with stimulation of forelimb cutaneous afferents. Additionally, although several studies have shown changes in cutaneous reflexes during locomotion after neurologic injury (Jones and Yang, 1994; Zehr et al., 1998; Frigon and Rossignol, 2006, 2007, 2008b; Frigon et al., 2009; Duysens et al., 2010; Zehr and Loadman, 2012), whether responses evoked by stimulating arm/forelimb and leg/hindlimb cutaneous afferents maintain their corrective or preventive functions, is less well understood. For example, stroke subjects show different kinematic changes at the knee and ankle joints with stimulation of the SP nerve compared with healthy subjects (Zehr et al., 1998). However, in that study, the two groups walked at different treadmill speeds (slower in stroke subjects) and were not age-matched (older in stroke subjects). We are currently investigating whole-body functional responses before and after incomplete spinal cord injury during locomotion in the same cats.

## Synthesis

Reviewing Editor: David Franklin, Technische Universitat Munchen Decisions are customarily a result of the Reviewing Editor and the peer reviewers coming together and discussing their recommendations until a consensus is reached. When revisions are invited, a fact-based synthesis statement explaining their decision and outlining what is needed to prepare a revision will be listed below. The following reviewer(s) agreed to reveal their identity: NONE.

Your manuscript has been reviewed by one expert in the field and by myself. We both have interest in the manuscript and think that this manuscript provides important insight into the coordination of the limbs during quadrupedal walking. We both appreciate the study design and high quality of data that examines the effect of peroneal nerve stimulation at different time points within the step cycle. However, we both have concerns about the statistics, data presentation, and the discussion of the material with related research in the field. The total volume of data presented is extensive and often difficult to see exactly what is critical for the study and the interpretation. Moreover, we both have concerns about the discussion and whether it places the current results into the broad literature in the area. Currently the discussion is mainly used to present the results again alongside the authors’ interpretation of the results. Instead, we believe that there is a strong need for the introduction and discussion to be revised to position these findings within the context of the literature and to provide a context for the importance of the work. However, we both have sufficient interest to see a revised version of the paper. I encourage you to revise your manuscript after addressing each of the concerns raised.

General comments from the reviewers:

This study examines the influence on the cat walking pattern of a brief stimulus to the superficial peroneal (SP) nerve delivered at 4 points in the step cycle. Previously, stimulation of SP has been shown to induce the stumbling corrective response in cats and humans, which has been characterized by the events and reflex responses evoked in the stimulated leg. This study asks what happens to the pattern of walking in all four legs of the quadruped. The primary finding is that the most impactful influences on the stepping pattern affect the hind limbs, both ipsilateral and contralateral to the stimulus, and primarily of the step within which the stimulus is delivered. There were some adaptations or adjustments noted in the step following the stimulus, but these were fewer and less robust. The study includes a very large amount of data from an extensive body of outcome measures. The study design is quite straight forward and executed well. However, the shear volume of data is overwhelming. The Conclusions are quite limited as the authors have elected to spend much of the Discussion retelling the Results and then adding their interpretation, with little integration of the existing literature or how their findings contribute to the understanding of the control of locomotion.

Major concerns:

The sample size is really quite small and it makes the use of parametric statistics quite risky. In addition, the authors of have normalized their EMG amplitude data to the control value, but have included the control value as one of the levels in their ANOVA. This artificially introduces a zero variance into the ANOVA which ...

The volume of data is overwhelming and distracting from the main message of the manuscript. The figures are incredibly dense and the data points, as a result, are very condensed making it challenging to work through the volume and try to gain meaning. The authors have recorded a large number of outcome variables, but have not clearly indicated which outcomes measures will actually be relevant to addressing the research question. Moreover, the research question itself is incredibly vague and open-ended. This has created the challenge in then reporting the data. It would be very useful if the authors could provide a clear hypothesis to their study and indicate which outcome measures will be specifically used to test the hypothesis. Surely, there is sufficient evidence in the literature to predict some, if not most, of the timing effects on the stimulated step cycle of the ipsilateral leg. This then must allow for logical prediction of outcomes for the other legs. And if the temporal and spatial pattern of the gait cycle is the primary outcome of interest, then the EMG data really are superfluous and add very little to the paper. This is particularly the case as there are significant gaps in the EMG profiles available because of the desire to record from all four limbs. For example, there are no pure flexors from the hind legs in the available data which will make it challenging to then use EMG patterns to link these data to the CPG literature arising from decerebrate or fictive preparations. My strong suggestion would be to focus the paper to a limited few outcome measures that will clearly address the research question. The additional data can certainly be used to corroborate the key findings, but should then be reduced in their prominence in this paper.

The Discussion lacks integration of this study’s findings with the extant literature. I appreciate that the effect of SP nerve stimulation on the other legs has not been described before. However, the influence of sensory stimuli, including of cutaneous afferent inputs, has been extensively studied in the context of the rhythmic control of stepping. This study clearly builds from the work and extends it and should be positioned within the literature in that way. The current Discussion is largely a retelling of the Results with the authors’ interpretation of the functional relevance, with little support for this interpretation arising from the literature and little integration of this interpretation into the theoretical framework of the neural control of locomotion. The Discussion needs extensive revision.

Other Comments

line 33: should read “Proprioceptive and tactile feedback regulates the stepping pattern ...”

Line 46-47: The introduction of the phrases Ipsilateral, Contralateral, Homolateral, and Diagonal limbs was confusing. It was not until I dug into the figures that the use of these terms made logical sense. Perhaps explain why these terms are specifically used at the time they are introduced i.e. why is it important to label the ipsilateral forelimb the homolateral limb - I get it now, but it confused me for a while.

Line 80: How long after the implant surgery did the recording session occur? Were all recordings made in a single experimental session? Were the cats only used for this study, or were these animals used in other studies? I ask because it is a bit concerning if these animals were used only for the collection of ∼60 stimuli with five to six steps between or roughly 10 min of walking. If the animals were used in other studies, it would be important to note that and to describe where within the timeline of the other studies these specific experiments occurred.

Line 85: presumably a portion of the Teflon coating was removed from the portion of the Cooner wire that was within the muscle belly

Line 86: It would be useful to describe how the connectors were mounted to the head

Line 87: should read “... electrode placement by electrically stimulating...”

Line 89-94: It would be useful to provide some information as to which cats contributed to the various combinations of muscles. The n for each muscle is described later, but it is not clear which muscles ended up being paired from the same animal. This is somewhat important if muscle activity is going to be used to interpret neural control i.e. in relation to concepts of CPG control and flexor or extensor phases etc. However, this is less relevant if EMG becomes a secondary dataset to corroborate the behavioral outcome measures. I would suggest that in the absence of a pure flexor from the hind legs, an in depth discussion of neural control mechanisms will not be able to be based EMG findings. It is also curious that TA was not recorded. I understand that later in the paper it is noted that stable TA EMG activity could not be recorded. However, TA is not a challenging muscle from which to record.

Line 107: should read “... stimulated the left or right SP nerve while walking at a speed between 0.3 m/s to 0.5 m/s.”

Line 110-111: If motor threshold is defined as evoking flexion response in the ipsilateral leg and a stimulus of 1.2 this threshold is used, then presumably this stimulus is recruiting flexor reflex afferents and not low threshold cutaneous afferents. This is very different from the stimulus intensities used in the studies that were cited in the background literature and Discussion, particular the link to the human work e.g. Zehr. This also would then significantly impact the interpretation of the findings as the flexor reflex is functionally quite different from the interlimb cutaneous reflexes or the stumbling corrective response.

Line 113: 25 pulses of 0.2 ms duration with an interpulse period of about 3.3 ms results in a stimulus of about 88 ms in duration. This is certainly longer than the 3 pulse train used in other studies. However, it would be helpful to provide a justification for this specific duration of stimulus. How does this relate to ecologically valid stimuli?

Line 162: EMG amplitude is normalized. It is never detailed how. We learn in Figure 10 from the y-axis label that the data are normalized to the control amplitude, but there is no description of this in the text. It also needs to be clarified how the control data were included in the parametric analysis of amplitude when the control amplitudes were artificially given zero variance. How was this handled within your analysis of variance?

Line 191: this is a bit disingenuous. From the figures, it cannot be clearly seen what changes actually occurred given the scale of the data. That notwithstanding, there are other obvious impacts aside from the inhibition in SOL, not the least of which is a burst in SRT in both 2A and D, which is also paired with an obvious prolongation of SRT duration in 2D. Moreover, knee and ankle angles, as well as toe height, are also clearly impact by the stimulus in 2A and D. Perhaps it would be more appropriate to simply state that there are clear differences in the responses evoked depending upon where within the step cycle the stimulus is delivered.

Line 214: should read “We generally find eight individual...” and similarly in line 216, the 9 should be written as nine.

Line 239: should read “... not affect step or stride lengths of any of the four limbs...”

Line 390: The increase in step length does not indicate a wider base of support, but a longer base of support. The authors cannot say anything about the width of the base of support as the only data they available are from the sagittal plane. They also cannot suggest that this longer base of support possibly increases stability as increase the base length with the same base width would be potentially destabilizing in the frontal plane. The authors should steer away from discussing stability without data from the frontal plane.

## Author Response

Author response: We thank the Reviewer and Editor for their constructive comments. We made substantial revisions. Below you will find answers to the comments. We hope that our revision is satisfactory.

Best regards,

The authors

Synthesis of Reviews:

Computational Neuroscience Model Code Accessibility Comments for Author (Required):

N/A

Synthesis Statement for Author (Required):

Your manuscript has been reviewed by one expert in the field and by myself. We both have interest in the manuscript and think that this manuscript provides important insight into the coordination of the limbs during quadrupedal walking. We both appreciate the study design and high quality of data that examines the effect of peroneal nerve stimulation at different time points within the step cycle. However, we both have concerns about the statistics, data presentation, and the discussion of the material with related research in the field. The total volume of data presented is extensive and often difficult to see exactly what is critical for the study and the interpretation. Moreover, we both have concerns about the discussion and whether it places the current results into the broad literature in the area. Currently the discussion is mainly used to present the results again alongside the authors’ interpretation of the results. Instead, we believe that there is a strong need for the introduction and discussion to be revised to position these findings within the context of the literature and to provide a context for the importance of the work. However, we both have sufficient interest to see a revised version of the paper. I encourage you to revise your manuscript after addressing each of the concerns raised.

General comments from the reviewers:

This study examines the influence on the cat walking pattern of a brief stimulus to the superficial peroneal (SP) nerve delivered at 4 points in the step cycle. Previously, stimulation of SP has been shown to induce the stumbling corrective response in cats and humans, which has been characterized by the events and reflex responses evoked in the stimulated leg. This study asks what happens to the pattern of walking in all four legs of the quadruped. The primary finding is that the most impactful influences on the stepping pattern affect the hind limbs, both ipsilateral and contralateral to the stimulus, and primarily of the step within which the stimulus is delivered. There were some adaptations or adjustments noted in the step following the stimulus, but these were fewer and less robust. The study includes a very large amount of data from an extensive body of outcome measures. The study design is quite straight forward and executed well. However, the shear volume of data is overwhelming. The Conclusions are quite limited as the authors have elected to spend much of the Discussion retelling the Results and then adding their interpretation, with little integration of the existing literature or how their findings contribute to the understanding of the control of locomotion.

Author response: We added several sentences to the introduction and discussion to integrate our results with the literature.

Major concerns:

The sample size is really quite small and it makes the use of parametric statistics quite risky. In addition, the authors of have normalized their EMG amplitude data to the control value, but have included the control value as one of the levels in their ANOVA. This artificially introduces a zero variance into the ANOVA which ...

Author response: We redid the statistical analysis with Friedman’s test (non-parametric). In the original manuscript, the statistical analysis of EMG amplitude were done on raw data and not on the normalized data. We now indicate this in the methods (lines 227-233 of tracked changes document).

The volume of data is overwhelming and distracting from the main message of the manuscript. The figures are incredibly dense and the data points, as a result, are very condensed making it challenging to work through the volume and try to gain meaning. The authors have recorded a large number of outcome variables, but have not clearly indicated which outcomes measures will actually be relevant to addressing the research question. Moreover, the research question itself is incredibly vague and open-ended. This has created the challenge in then reporting the data. It would be very useful if the authors could provide a clear hypothesis to their study and indicate which outcome measures will be specifically used to test the hypothesis. Surely, there is sufficient evidence in the literature to predict some, if not most, of the timing effects on the stimulated step cycle of the ipsilateral leg. This then must allow for logical prediction of outcomes for the other legs. And if the temporal and spatial pattern of the gait cycle is the primary outcome of interest, then the EMG data really are superfluous and add very little to the paper. This is particularly the case as there are significant gaps in the EMG profiles available because of the desire to record from all four limbs. For example, there are no pure flexors from the hind legs in the available data which will make it challenging to then use EMG patterns to link these data to the CPG literature arising from decerebrate or fictive preparations. My strong suggestion would be to focus the paper to a limited few outcome measures that will clearly address the research question. The additional data can certainly be used to corroborate the key findings, but should then be reduced in their prominence in this paper.

Author response: We disagree that all research must be hypothesis driven. However, we agree that clear predictions can be made and we added several sentences to the introduction. We removed one figure as well as the second cycle post-stimulation, which lightens the figures and result. The EMG data were used to determine if they can explain spatiotemporal adjustments in the gait pattern. Although there may be gaps in the EMG profiles, the number of recordings we have is considerably higher than the majority of studies and we believe that these data are valuable. We are currently rethinking how we present EMG data in our papers. Pure flexors in the hindlimbs are rare, except for the iliopsoas and tibialis anterior. The knee has no pure flexors. If our goal was to link our data with existing CPG literature, then the sartorius anterior burst is representative of the flexion phase, displaying a similar burst as TA, EDL and iliopsoas (see studies by McCrea, Rossignol, etc.). For an example, see Figure 3 of Pearson and Rossignol (1991, J Neurophysiol 66(6): 1874-87).

The Discussion lacks integration of this study’s findings with the extant literature. I appreciate that the effect of SP nerve stimulation on the other legs has not been described before. However, the influence of sensory stimuli, including of cutaneous afferent inputs, has been extensively studied in the context of the rhythmic control of stepping. This study clearly builds from the work and extends it and should be positioned within the literature in that way. The current Discussion is largely a retelling of the Results with the authors’ interpretation of the functional relevance, with little support for this interpretation arising from the literature and little integration of this interpretation into the theoretical framework of the neural control of locomotion. The Discussion needs extensive revision.

Author response: We agree and we added several sentences to the discussion (see tracked changes document).

Other Comments

line 33: should read “Proprioceptive and tactile feedback regulates the stepping pattern ...”

Author response: Changed to: Proprioceptive and tactile feedback regulates the locomotor pattern ...

Stepping during locomotion is redundant.

Line 46-47: The introduction of the phrases Ipsilateral, Contralateral, Homolateral, and Diagonal limbs was confusing. It was not until I dug into the figures that the use of these terms made logical sense. Perhaps explain why these terms are specifically used at the time they are introduced i.e. why is it important to label the ipsilateral forelimb the homolateral limb - I get it now, but it confused me for a while.

Author response: This is a good suggestion. We changed the sentence to:

Stimulating nerves of the foot, such as the superficial peroneal (SP) nerve that innervates the foot dorsum, elicits specific reflex responses in the ipsilateral (stimulated limb) and contralateral (opposite limb of same girdle) legs/hindlimbs but also in the homolateral (limb of the other girdle on same side) and diagonal (limb of the other girdle on opposite side) arms/forelimbs in cats and humans during locomotion (Haridas and Zehr, 2003; Hurteau et al., 2018; Pearcey and Zehr, 2019).

We also added a diagram to Figure 1 (1C).

Line 80: How long after the implant surgery did the recording session occur? Were all recordings made in a single experimental session? Were the cats only used for this study, or were these animals used in other studies? I ask because it is a bit concerning if these animals were used only for the collection of ∼60 stimuli with five to six steps between or roughly 10 min of walking. If the animals were used in other studies, it would be important to note that and to describe where within the timeline of the other studies these specific experiments occurred.

Author response: The use of animals for other studies was mentioned in the last section of the discussion. We added the following sentences to the first section of the methods (lines 98-108 of tracked changes document):

As part of our effort to maximize the scientific output of each animal, all animals participated in other studies to answer other scientific questions, some of which have been published (Lecomte et al., 2021). For instance, cats participated in experiments where they walked on a split-belt treadmill at different speeds and left-right speed differences with or without stimulating peripheral nerves, such as SP, superficial radial and distal tibial. They also performed quadrupedal and hindlimb-only locomotion in the forward and backward directions (Harnie et al., 2022, 2021). Cats also stepped on a walkway where they negotiated obstacles and stepped along a circular path. In all seven cats of the present study, we performed a lateral spinal hemisection at mid-thoracic levels and performed the same data collection as in the intact state. We collected the data presented here three to six weeks after implanting electrodes during a single experimental session.

Line 85: presumably a portion of the Teflon coating was removed from the portion of the Cooner wire that was within the muscle belly. Line 86: It would be useful to describe how the connectors were mounted to the head.

Author response: The following sentences were modified/added:

To record EMG, we directed pairs of Teflon-insulated multistrain fine wires (AS633; Cooner Wire) subcutaneously from two head-mounted 34-pin connectors (Omnetics) that were sewn into the belly of selected fore- and hindlimb muscles for bipolar recordings, with 1-2 mm of insulation stripped from each wire. The head connector was secured to the skull using dental acrylic and four to six screws.

Line 87: should read “... electrode placement by electrically stimulating...”

Author response: Yes, this has been corrected.

Line 89-94: It would be useful to provide some information as to which cats contributed to the various combinations of muscles. The n for each muscle is described later, but it is not clear which muscles ended up being paired from the same animal. This is somewhat important if muscle activity is going to be used to interpret neural control i.e. in relation to concepts of CPG control and flexor or extensor phases etc. However, this is less relevant if EMG becomes a secondary dataset to corroborate the behavioral outcome measures. I would suggest that in the absence of a pure flexor from the hind legs, an in depth discussion of neural control mechanisms will not be able to be based EMG findings. It is also curious that TA was not recorded. I understand that later in the paper it is noted that stable TA EMG activity could not be recorded. However, TA is not a challenging muscle from which to record.

Author response: We added a Table (Table 1). There are very few pure flexors in the hindlimbs, iliopsoas for the hip and tibialis anterior (TA) for the ankle, with none at the knee. We always implant TA and it is not a challenging muscle to implant or record but we rarely get good recordings, for reasons we cannot explain. In some cats, we get a flexor burst, in other cats we get a flexor and a stance-related burst and in other cats, we obtain mainly an extensor-related burst. Consequently, we cannot say if the different burst profiles are due to individual gait strategies or to issues with recordings. During fictive locomotion in the cat, TA and EDL have the same burst profiles as SRT and iliopsoas. Bursts in posterior biceps with (PBST) or without ST during fictive locomotion depend on the preparation (e.g. spontaneous vs MLR-evoked fictive locomotion, spinal vs spinal-intact). The EMG data set in the present study are mainly to corroborate the behavioral outcome measures.

Line 107: should read “... stimulated the left or right SP nerve while walking at a speed between 0.3 m/s to 0.5 m/s.”

Author response: Sentence changed to:

During experiments, we stimulated the left or right SP nerve while cats stepped at speeds of 0.3 m/s to 0.5 m/s.

Author response: When possible, we prefer to avoid the term ‘walking’ because the walk is a type of quadrupedal gait, which may or may not be used.

Line 110-111: If motor threshold is defined as evoking flexion response in the ipsilateral leg and a stimulus of 1.2 this threshold is used, then presumably this stimulus is recruiting flexor reflex afferents and not low threshold cutaneous afferents. This is very different from the stimulus intensities used in the studies that were cited in the background literature and Discussion, particular the link to the human work e.g. Zehr. This also would then significantly impact the interpretation of the findings as the flexor reflex is functionally quite different from the interlimb cutaneous reflexes or the stumbling corrective response.

Author response: We prefer to avoid the term flexor reflex afferent. It is not possible to selectively activate high-threshold afferents that generate the withdrawal reflex without activating low-threshold afferents that generate the stumbling corrective reaction. We are not aware of studies that have demonstrated shared or distinct neuronal circuits between the two types of reflexes. It is likely that they share several components. Our results are not consistent with activation of high threshold afferents because this would have generated a flexor withdrawal reflex throughout the locomotor cycle, in both swing and stance (see Forssberg 1979). We added the following sentences to the discussion (lines 451-457 of tracked changes document):

At this stimulation intensity, it is unlikely that we activated high threshold nociceptive afferents that mediate the withdrawal reflex because such activation elicits flexion withdrawal throughout the locomotor cycle, including during the stance phase as well as during quiet standing (Buford and Smith, 1993; Forssberg, 1979; Zehr et al., 1997). Moreover, cats did not display discomfort with stimulation. Thus, it is likely that temporal summation of inputs from low-threshold SP afferents evoked the large response observed with stimulation at mid-swing and at the stance-to-swing transition.

Line 113: 25 pulses of 0.2 ms duration with an interpulse period of about 3.3 ms results in a stimulus of about 88 ms in duration. This is certainly longer than the 3 pulse train used in other studies. However, it would be helpful to provide a justification for this specific duration of stimulus. How does this relate to ecologically valid stimuli?

Author response: We used stimulation parameters similar to those used by Quevedo et al. (2005a,b) to investigate the neuronal circuits involved in the stumbling corrective reaction with SP nerve stimulation. We are not sure how this relates to ecologically valid stimuli. This is difficult to assess because when the cat’s foot dorsum makes contact with an obstacle, hair cells are first deflected followed by other mechanoreceptors in superficial and deep layers of the skin. The stumbling corrective reaction can result from a combined activation of these different receptors. We added the following sentences to the methods (lines 160-168 of tracked changes document):

We determined motor threshold during mid-swing and used the same stimulation intensity in the other phases. Each stimulation consisted of a train of 25 impulses (0.2 ms duration, 300 Hz frequency) for a total train duration of 88 ms. These stimulation parameters are similar to those used to describe the neuronal circuits involved in the stumbling corrective reaction with SP nerve stimulation in decerebrate curarized cats during fictive locomotion (Quevedo et al., 2005a, 2005b). The correspondence of these stimulation parameters to natural stimuli is not easy to demonstrate because when the cat’s foot dorsum contacts an obstacle, hair cells are first deflected followed by mechanoreceptors in superficial and deep layers of the skin. The stumbling corrective reaction can result from a combined activation of these different receptors. However, Buford and Smith reported that the foot dorsum made contact for ∼100 ms with the force-sensitive rod, which is close to our 88 ms train.

Line 162: EMG amplitude is normalized. It is never detailed how. We learn in Figure 10 from the y-axis label that the data are normalized to the control amplitude, but there is no description of this in the text. It also needs to be clarified how the control data were included in the parametric analysis of amplitude when the control amplitudes were artificially given zero variance. How was this handled within your analysis of variance?

Author response: This was our mistake and it is now clarified in the text. The normalization was/is for illustrative purposes only. We performed the statistical analysis on raw data. We added the following sentence (lines 219-222 of tracked changes document):

For illustrative purposes, EMG amplitudes are normalized to the control amplitude (i.e., amplitude measured during the cycle preceding stimulation) and expressed as a percentage. However, we performed the statistical analysis of the EMG amplitude on the raw data.

Line 191: this is a bit disingenuous. From the figures, it cannot be clearly seen what changes actually occurred given the scale of the data. That notwithstanding, there are other obvious impacts aside from the inhibition in SOL, not the least of which is a burst in SRT in both 2A and D, which is also paired with an obvious prolongation of SRT duration in 2D. Moreover, knee and ankle angles, as well as toe height, are also clearly impact by the stimulus in 2A and D. Perhaps it would be more appropriate to simply state that there are clear differences in the responses evoked depending upon where within the step cycle the stimulus is delivered.

Author response: Yes, we added some text and the suggested sentence. See lines 249-258 of tracked changes document.

Line 214: should read “We generally find eight individual...” and similarly in line 216, the 9 should be written as nine.

Author response: Changed as suggested.

Line 239: should read “... not affect step or stride lengths of any of the four limbs...”

Author response: Changed as suggested.

Line 390: The increase in step length does not indicate a wider base of support, but a longer base of support. The authors cannot say anything about the width of the base of support as the only data they available are from the sagittal plane. They also cannot suggest that this longer base of support possibly increases stability as increase the base length with the same base width would be potentially destabilizing in the frontal plane. The authors should steer away from discussing stability without data from the frontal plane.

Author response: The reviewer is correct. This was removed from the revised manuscript.
